# Current Source Density Analysis of Electroantennogram Recordings: A Tool for Mapping the Olfactory Response in an Insect Antenna

**DOI:** 10.3389/fncel.2018.00287

**Published:** 2018-09-05

**Authors:** Vincent E. J. M. Jacob

**Affiliations:** CIRAD, UMR PVBMT, St. Pierre, La Réunion, France

**Keywords:** insect, olfaction, electroantennogram, olfactory receptor neurons, antenna, current source density analysis

## Abstract

The set of chemosensory receptors expressed by the olfactory receptor neurons lying in an insect's antennae and maxillary palps define the ability of this insect to perceive the volatile chemicals of its environment. The main two electrophysiological methods of antennal recordings for studying the range of chemicals that activate chemosensory receptors have limitations. Single-sensillum recording (SSR) samples a subset of olfactory receptor neurons and therefore does not reveal the full capacity of an insect to perceive an odor. Electroantennography (EAG), even if less resolutive than SSRs, is sometimes preferred since it samples the activity of a large number of the olfactory receptor neurons. But, at least in flies, the amplitude of the EAG signal is not directly correlated with the degree of sensitivity of the insect to the olfactory compound. Such dual methodology was also used to study mammalian brains, and the current source density (CSD) analysis was developed to bridge the gap between the cellular and the population recordings. This paper details the use of a similar approach adapted to the study of olfactory responses within insects with bulbous antennae. The EAG was recorded at multiple antennal positions and the CSD that generates the EAG potentials were estimated. The method measures the activation of olfactory receptor neurons (ORNs) across the antennae and thus it quantifies the olfactory sensitivity of the insect. It allows a rapid mapping of olfactory responses and thus can be used to guide further SSRs or to determine that two chemicals are detected by independent ORNs. This study further explored biases resulting from a limited number of recording positions or from an approximation of the antennal geometry that should be considered for interpreting the CSD maps. It also shows that the CSD analysis of EAGs is compatible with a gas chromatograph stimulator for analyzing the response to complex odors. Finally, I discuss the origin of the EAG signal in light of the CSD theory.

## Introduction

Insects live in an environment filled with numerous volatile compounds, but they detect and interact with only a few of them (Clavijo Mccormick et al., 2014) via their chemosensory receptors that includes olfactory receptors (ORs), ionotropic receptors (IRs) and gustatory receptors (GRs). The olfactory organs of the insects, antennae and maxillary palps, are covered with many olfactory sensilla, which house the dendrites of the olfactory receptor neurons (ORNs) (Shanbhag et al., 1999). The chemosensory receptors are located in the membrane of the ORN dendrites and are responsible for the activation of the ORNs by selective compounds (Joseph and Carlson, 2015).

Deciphering how insects perceive their chemical environment requires investigating the functions of the chemosensory receptors in the living insects. Two main types of antennal recordings were used over the past 60 years for this purpose (Olsson and Hansson, 2013): electroantennography (EAG) (Schneider, 1957a,b), which consists in recording field potentials across the antenna, and single-sensillum recording (SSR) (Einzelnen, 1962; Schneider and Boeckh, 1962), which consists in recording the spiking activity elicited by the ORNs of a single sensillum. SSR has some advantages over EAG. The signal is easier to interpret since it consists in the timing of the action potentials that travel through the antennal nerve and deliver the detected sensory information to antennal lobe neurons in the brain. Thus, SSR reliably measures the ORN output. Furthermore, some labeled-line behaviors are directly correlated with the activity level of a class of ORNs (Stensmyr et al., 2012; Dweck et al., 2013, 2015; Mansourian et al., 2016). However, the arbitrary sampling of individual sensilla provides an incomplete access to the functions of the olfactory organ. First, exploring the antennal responsiveness requires pooling the data from many individuals. Therefore, the interindividual variability cannot be estimated. Secondly, some classes of ORNs could be completely disregarded. An exhaustive exploration of the insect sensitivity to chemicals requires to sample all the different classes of chemosensory receptors expressed in an insect, and this information is solely available in *Drosophila* and demands significant experimental effort (Dweck et al., 2013, 2015, 2016; Nowotny et al., 2014; Ronderos et al., 2014; Münch and Galizia, 2016). Finally, the number of ORNs expressing the same chemosensory receptor is an important coding parameter that is difficult to estimate through SSR. It affects the latency of response and the sensitivity of second-order olfactory neurons (Bhandawat et al., 2007; Rospars et al., 2014).

Many researchers in chemical ecology use EAG rather than SSR because it provides a quick overview of the insect sensitivity to chemicals. The EAG signal results from the summation of the activity of many ORNs, thus combining different classes of chemosensory receptors. The amplitude of the EAG response depends both on the receptor potential response of individual ORNs (Kaissling, 1986; Lucas and Renou, 1992) and on the density of responsive ORNs in the vicinity of the recording electrode (Bigiani et al., 1989; Crnjar et al., 1989). It is tempting to consider that some evolutionary mechanisms should improve the detection of ecologically important chemicals through the involvement of more ORNs with an increased sensitivity, resulting in a large EAG response. However, experimental factors like the position of the recording electrode affects differently the amplitude of response to diverse chemicals (Bigiani et al., 1989; Crnjar et al., 1989; Biasazin et al., 2014; Jacob et al., 2017b). A small EAG response cannot be interpreted as a poor ability of the insect to detect a chemical. Obviously, neither SSR nor EAG are fully satisfying for quantitatively exploring the sensitivity of many insects to the constituents of their chemical environment.

The EAG is related to the local field potential (LFP) recordings in brain tissues that are known to depend on the complex properties of the propagation of electrical fields through the extracellular medium (Bédard and Destexhe, 2012). As in LFP, the contribution of an individual ORN to the EAG signal is inversely proportional to its distance to the recording electrode (Nagai, 1983b), so the EAG samples the activity of a subpopulation of antennal neurons. This consideration has a direct consequence for studying *Drosophila melanogaster* since morphological, functional and molecular observations over the last 20 years showed that each responsive class of ORN is confined in a sub-region of the funiculus (the third antennal segment; Couto et al., 2005) and this was also observed in other Muscomorpha species (Olsson et al., 2006; Tait et al., 2016). Clearly, an individual EAG recording misses or underestimates the response to some compounds in these species (Jacob et al., 2017b). Therefore, to estimate the sensitivity of an insect to olfactory compounds one must screen the antenna with varying the position of the EAG electrode (Biasazin et al., 2014; Jacob et al., 2017b). Since the pioneer work of Walter Pitts (Pitts, 1952), the methods of current source density (CSD) analysis (Nicholson, 1973; Mitzdorf, 1985; Buzsáki et al., 2012) have been developed to infer the single-cell activity from the population recordings. It consists in recording the LFPs at multiple positions and modeling the electrical field to localize the activated neurons (see Pettersen et al., 2006; Potworowski et al., 2012) for recent CSD models). In a recent paper we showed that CSD analysis can be applied to the EAG recorded in different Muscomorpha species and results in reproducible maps of antennal activation (Jacob et al., 2017b). This paper outlines a model of insect antenna used for analyzing the CSD from multiple EAG recordings. New experimental and modeled data are included here to enhance the interpretation of the CSD estimated by the model and to explore the limits of the method. CSD maps were found to correlate with response maps derived from ORN response data. However, an improper electrode sampling or a rough estimation of the antennal geometry were shown to bias the estimated CSD. As a proof of concept, example recordings are shown in several species of fruit flies and in the moth *Chilo sacchariphagus*, suggesting that CSD analysis can be used in various insect orders. In addition, the CSD maps were coupled with a gas chromatograph (GC), thus it is suited for future chemical ecology research and might be useful in the discovering of new attractants for insect pests.

## Material and methods

### Insects used in this study

EAG datas were collected on sexually mature females of the species *Zeugodacus cucurbitae, Ceratitis catoirii, Neoceratitis Cyanescens, Drosophila melanogaster*, and the moth *Chilo sacchariphagus*. Antenna were measured on *D. melanogaster* (standard wild-type laboratory strain Canton Special, CS), on *C. sacchariphagus*, and on six tephritid species, namely *Ceratitis capitata, C. catoirii, N. cyanescens, Bactrocera zonata, Z. cucurbitae*, and *Dacus demmerezi*. *C. capitata, C. catoirii*, and *B. zonata* were reared on artificial diet (Duyck and Quilici, 2002), as well as *D. melanogaster*. *N. cyanescens* was reared on potato (*Solanum tuberosum*), and *Z. cucurbitae* and *D. demmerezi* were reared on zucchini (*Cucurbita pepo*). A *C. sacchariphagus* larva was collected in a sugar cane field then reared and the adult was studied after emergence. Insects were reared at 25 ± 1°C and with 65 ± 10% relative humidity and a 12:12 h light:dark photoperiod.

### Odor delivery system

A humidified air stream (23 ml/s, air speed 60 cm/s), filtered through a charcoal filter, was continuously delivered to the insect antenna through a 7-mm glass tube held at 4 mm. Stimuli were applied by inserting a Pasteur pipette 15 cm upstream containing a small piece of filter paper loaded with 1 μl of a volatile compound diluted at 10^−2^ in paraffin oil. The compounds used in this paper were Z3-hexenyl acetate, linalool, methyl salicylate, ethyl butyrate or E2-hexenal. A puff of air (200 ms, 5 ml/s) was delivered through the pipette with an electro-valve (LHDA-1233215-H, Lee Company, France) controlled by a digital output module (NI 9472, National Instr., Nanterre, France) and the software Labview (National Instr.). A control pipette was loaded with 1 μl of paraffin oil. Control stimulations were applied twice, before and after a sequence of stimulation with each chemical applied in random order. Time intervals of 1 min were applied between consecutive puffs to limit the neuronal adaptation to chemicals.

Alternatively, the antenna was stimulated with a gas chromatograph (Clarus 580 GC, Perkin-Elmer, USA) injected with a mixture of Z3-hexenyl acetate, linalool, methyl salicylate, ethyl butyrate, pentyl acetate, ethyl acetate, and 1-octen-3-ol each diluted at 10^−3^ in hexane. The output capillary of the GC went through a 3 m transfer line (Antelia, Dardilly, France) heated at 250°C and its tip was inserted into the 7 mm glass tube instead of the Pasteur pipette.

### Electrophysiology

Insects were secured in a plastic tube, and the head was fixed with dental wax, leaving the antennae exposed. Both the reference and the recording electrode were glass capillary electrodes (tip diameter 1–2 μm, filled with 120 mM NaCl, 5 mM KCl, 1 mM CaCl_2_, 4 mM MgCl_2_, and 10 mM HEPES). The reference electrode was inserted in the right eye, and the recording electrode was leaned against the left antenna without insertion. The same procedure was used for the moth *C. sacchariphagus*. The EAG was recorded consecutively while displacing the recording electrode in *N* = 4 or 7 regularly interleaved positions, between 0 (adjacent to the basis of the arista in flies) and 1 (funiculus tip) along the proximo-distal axis. The recording position was set manually and was always on the middle axis of the lateral side of the funiculus. For the experiments with four antennal positions, the positions were explored in a random order. For the experiments with seven recording positions, the anatomical order (proximal to distal or distal to proximal) was used to assure that the spacing was as regular as possible. The signal was amplified (x200), low-pass filtered (1 kHz) with a DAGAN Ex-1 amplifier (Minneapolis, Minnesota, USA), and was digitized at 500 Hz (NI 9215, National instr.) with Labview software. For quantifying the EAG response amplitude, the EAG was filtered with a Gaussian convolution of 20 ms width, and response to control was subtracted. Amplitude was defined as the maximum negative peak in the 0.5 s following stimulation minus the average value in the 0.5 s preceding stimulation.

### Current source density (CSD) analysis

**Figure 1:**
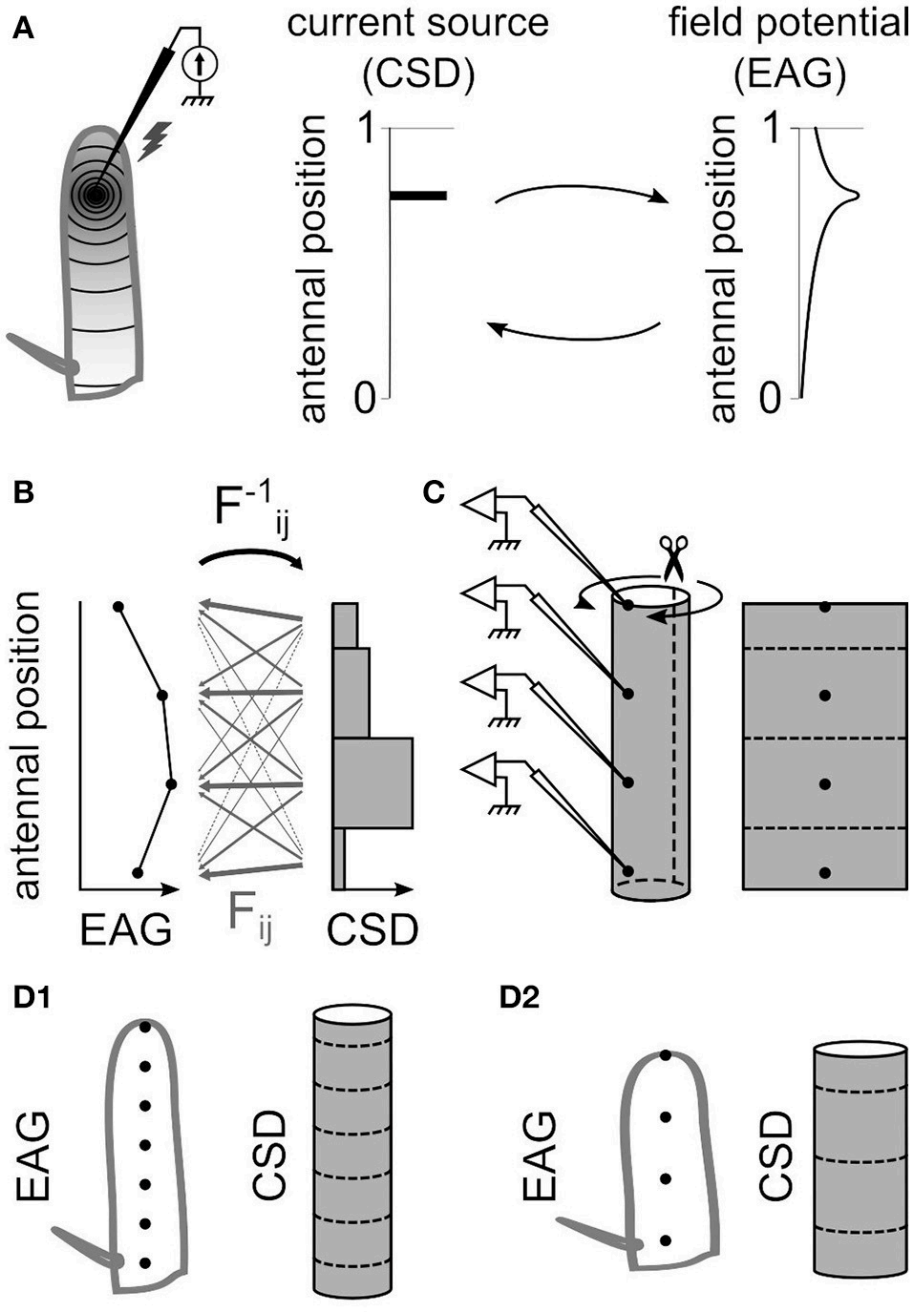
The antennal model for estimating current source density. **(A)** Electrostatic principles on the insect antenna. Left, a source of current at a given position on the funiculus is schematized. A static source of current at a specific antennal locus (middle) induces an electric field along the antenna that gets weaker with the distance from the source (right). The distribution of current sources and the distribution of potentials can be reciprocally deduced from each other. **(B)** For a model with four EAG positions, the CSD is estimated in four compartments. The EAG linearly depends on the CSD, and the 4 × 4 linear coefficients *F*_*ij*_ are symbolized with gray arrows. The thickness of each arrow symbolize the strength of the coefficient: it is small for the current sources far from the recording site, and it is large for the current sources close to the recording site. The reciprocal coefficients *F*^−1^_*ij*_ could be detailed in the same way. **(C)** The model of funiculus is a cylinder with the recording positions located on the surface (left). The current sources are on the surface only, and only the surface is considered conductive. Hence, the surface of the cylinder was virtually unfolded (right) and subdivided in the same number of compartments than the number of recording sites. **(D)** The dimensions of the cylinder that models the funiculus was different for each species. The figures show a model for an elongated funiculus with seven recording positions **(D1)** and for a globular funiculus with four recording positions **(D2)**.

The field potentials recorded in the neuronal tissues are caused by current sinks and sources surrounding the activated neurons. Current sources correspond to positive electric charges while current sinks correspond to negative charges. In the antennal surface, the activation of the ORNs result in a current sink near the dendrites that induce a negative potential recorded by the EAG electrode (Figure 10; Kaissling, 1986, 1995). The location of the current sinks in an insect antenna, and therefore of the activated ORNs, can be estimated from the spatial distribution of field potentials on the antennal surface (Figure 1A). To do so, the method used in this paper was adapted from the inverse method proposed by Pettersen and colleagues (Pettersen et al., 2006). It consists in three steps: (1) calculate the linear function that transforms a given distribution of CSD into the resulting spatial distribution of EAGs, (2) deduce the inverse linear function that transforms a given distribution of EAGs into the CSD it originates from, and (3) apply the inverse function to recorded EAGs.

The spatial distribution of potentials is estimated from EAGs recorded at *N* positions on the antenna (*N* = 4 or 7), and accordingly the current sources were estimated in *N* compartments, each compartment corresponding to the antennal surface surrounding one recording position. The CSD distribution is approximated with the hypothesis that the CSD is constant on each compartment. This assumption is necessary to get a unique solution of CSD distribution from a given distribution of EAGs, but may result in potential biases addressed in the result section. Intuitively, the EAG recorded at a position on the antenna depends strongly of the nearby current sources, and weakly on the distant ones. For each antennal position *x*_*i*_, the EAG potential recorded at this position was noted ϕ_*i*_ and the current source around this position was noted *C*_*i*_. Each antennal compartment contributes additively to the potential ϕ_*i*_ as written in the Equation (1).

(1) $$\phi_{i}=\sum_{j}F_{ij}{}^{*}\;C_{j}$$

The linear coefficients *F*_*ij*_ describe the influence of the current source at the position *x*_*j*_ to the EAG recorded at *x*_*i*_, and decreases with the distance between *x*_*i*_ and *x*_*j*_. In matrix formulation:

(2) $$\begin{array}{r} \phi =F{\;}^{*}\;C. \end{array}$$

The next section explains how to calculate directly the coefficients *F*_*ij*_ trough the electrostatic forward solution applied to a model of antenna. Assuming constant electrical conductivity, a point source current *I*_c_ spreads uniformly in all directions and generates a potential ϕ at distance *r* from the source given by Equation (3) where σ is the conductivity of the medium.

(3) $$\begin{array}{r} \phi \;=\;I_{\text{c}}/4\pi \sigma r \end{array}$$

A uniform current source with any geometrical shape can be considered as the sum of the point source currents that constitute the shape. How it affects the electric potential at a given position and distance can then be estimated by integrating spatially Equation (3). This general framework from Pettersen et al. (2006) was applied to the specific geometry of the antennal model. The geometry of the antenna was approximated with a cylinder (Figure 1C). With such approximation, to model can be applied to different insects with minimal measurements of the antennal dimensions. As an alternative, cone-like shapes were tested with varying the diameter of each compartment. The current sinks are generated by the dendritic activity of the ORNs located on the surface of the funiculus, so in our model they are located on the external surface of the cylinder. Because the amplitude of the signal drops as soon as the electrode penetrates the cuticle, the electric conductivity in the internal medium was assumed to be low compared to the conductivity on the antennal surface and was neglected. Therefore, the electric field propagates only through the surface of the funiculus. Thus, a simplified model was used with a 2D geometry corresponding to a virtual unfolding of the funiculus surface. Each current source was assumed to be uniform over a rectangular area surrounding an electrode position. The current sources were estimated at *N* spatial positions, *N* being the number of recording positions. Given this geometry and Equation (3), the potential ψ_*ij*_ generated at electrode *x*_*j*_ by current source *C*_*i*_ around the electrode position *x*_*i*_ is:

(4) $$\begin{array}{r} \psi_{ij}=\frac{1}{4\pi \sigma }\overset{q}{\underset{-q}{\int }}\overset{x_{i}+\frac{h}{2}}{\underset{x_{i}-\frac{h}{2}}{\int }}\frac{1}{\sqrt{{\left(x-x_{j}\right)}^{2}+y^{2}}}dxdy*C_{i} \end{array}$$

(5) $$\begin{array}{r} F_{ij}=\frac{1}{4\pi \sigma }\overset{q}{\underset{-q}{\int }}\overset{x_{i}+\frac{h}{2}}{\underset{x_{i}-\frac{h}{2}}{\int }}\frac{1}{\sqrt{{\left(x-x_{j}\right)}^{2}+y^{2}}}dxdy \end{array}$$

where *q* is the width of the rectangular current source (circumference of the funiculus cross-section), and *h* is the spacing between electrodes. The parameters q and h were estimated for each species (Figure 1D) by measuring the size of the funiculus of the left antenna (length, width, and thickness) with a light microscope; the average of 10 individuals was used for each species. Wherever unmentioned, the dimensions of *Z. cucurbitae* antennae were used. The circumference of the funiculus cross section was estimated from the approximation of Ramanujan (1914) for circumference of an ellipse $\pi *\left(3\left(a+b\right)-\sqrt{\left(3a+b\right)\left(a+3b\right)}\right)$, with *a* and *b* being the two radius of the ellipse (funiculus width/2 and funiculus thickness/2). The conductivity σ was measured in 11 individuals with using two electrodes simultaneously on the antennal surface. In Equations (4,5), the median value σ = 10 mΩ/mm (25–75 percentiles: 7–16 mΩ/mm) was used. The parameter σ has a multiplicative effect thus does not affect the relative amplitudes of the CSD, and in particular the spatial distribution of CSD is unaffected. Corrections on Equations (4,5) were made for current sources at both extremities of the funiculus. At the distal end *x*_*N*_, the electrode is located at the tip of the funiculus so that there is no neuron beyond the electrode position. Thus, *dx* varied between *x*_*N*_ – *h*/2 and *x*_*N*_. Scanning electron microscopy experiments revealed that for the insect species of this paper, no olfactory sensilla lied more proximal than the arista where the electrode at the proximal end *x*_1_ was located; as a consequence, *dx* ranged from *x*_1_ to *x*_1_ + *h/2*. When a CSD model was adapted to a subset of the electrodes, the two outward electrodes were not necessarily located at the extremities of the funiculus. In such case the most proximal and most distal CSD compartments were extended up to the arista and to the distal tip of the funiculus with adjusting the integration window.

The coefficients *F*_*ij*_ were calculated directly from Equation (5). Reciprocally, the CSD in each compartment is a linear combination of the *N* amplitudes of EAG response (Figure 1B). The linear coefficients *F*^−1^_*ij*_ for estimating the CSD distribution from the EAG signals can be directly calculated as the inverse of the matrix *F*. The best estimate of current sources *C*_*i*_ when the potentials ϕ _*j*_ are known is then given by the reverse formula:

(6) $$\begin{array}{r} C\;=\;F^{-1}{\;}^{*}\;\phi \end{array}$$

(7) $$\begin{array}{r} C_{i}\;=\;\sum_{j}F_{ij}^{-1}{\;}^{*}\;\phi_{j} \end{array}$$

Mathematically, the spatial distribution of CSD amounts to a representation of the distribution of EAG signals observed through a change of coordinates. The CSD was estimated at each time point after stimulation. The area of the CSD response was quantified by reversing and integrating the CSD signal between 0 and 1.5 s after stimulation, and the amplitude of the CSD response was defined as the minimal CSD signal in the 500 ms interval preceding the stimulation minus the minimal CSD signal in the 500 ms interval following the stimulation. Without further indication, CSD response refers to the area of the CSD response in the text. The antennal activation was localized with the center of mass of the positive CSD responses, defined as the sum of response × position divided by the sum of responses.

### Cellular functional maps in *D. melanogaster*

One-dimensional functional maps of *D. melanogaster* antenna in response to a given compound were estimated from the response levels of each basiconic and trichoid classes of antennal ORNs, the number sensilla in each class on a female *D. melanogaster* antenna, and their spatial distribution on the proximo-distal axis. The response levels of the antennal ORNs to methyl salicylate, linalool, ethyl acetate, 1-octen-3-ol, pentyl acetate and ethyl butyrate were extracted from the database DoOR (Münch and Galizia, 2016). The database didn't include the response to at1 ORNs, but small esters and polar compounds probably induce little response in these ORNs. The responses to coeloconic sensilla were not considered either but, since these sensilla are low in number and sparsely distributed, their contribution to the antennal responsiveness should be small. The number of sensilla per classes were reported in Grabe et al. (2016), which amount to 8 ab3, 39.825 ab1, 23 ab2, 14 ab4, 15 ab6, 34 ab5, 11.25 ab7, 18 ab8, 18 ab10, 24 ab9, 62.5 at1, 27 at3, 15 at2, and 19.5 at4. The spatial distributions of the different sensilla types on *D. melanogaster* antenna were estimated from de Bruyne et al. (2001), Grabe et al. (2016), Lin and Potter (2015), and parametrized as follow. The antennal proximo-distal position varied between 0 (departure of the arista) to 1 (tip of the funiculus). For each sensilla type, the proximo-distal spatial distribution was approximated by logit normal distributions that are bounded on the [0,1] interval (Figure 3B). For basiconic sensilla, the centers of the distributions were regularly interleaved from 0.1 for ab1 to 0.85 for ab9 and had a standard deviation of 0.1, with the exception of ab3 sensilla, the distribution of which was centered on 0.05 and had a standard deviation of 0.05. For trichoid sensilla, the logit normal distributions were centered on positions 0.65, 0.7, 0.75, and 0.8 and had a standard deviation of 0.15. As an alternative, Gaussian distribution were tested and resulted in the same qualitative result. The distributions were expressed in sensilla density (number of sensilla per unit of antennal length). The response density was defined as the sum of the responses of each ORN multiplied by the corresponding number of sensilla and multiplied by the corresponding spatial distribution. In addition, 1,000 simulations of response density were obtained with attributing a random level of activation to each basiconic sensilla. The simulated response densities were used to calculate EAGs using a CSD model with 100 compartments and with the hypothesis that the responses densities are current sources. CSD were estimated backward using a model with four compartments.

## Results

**Figure 2:**
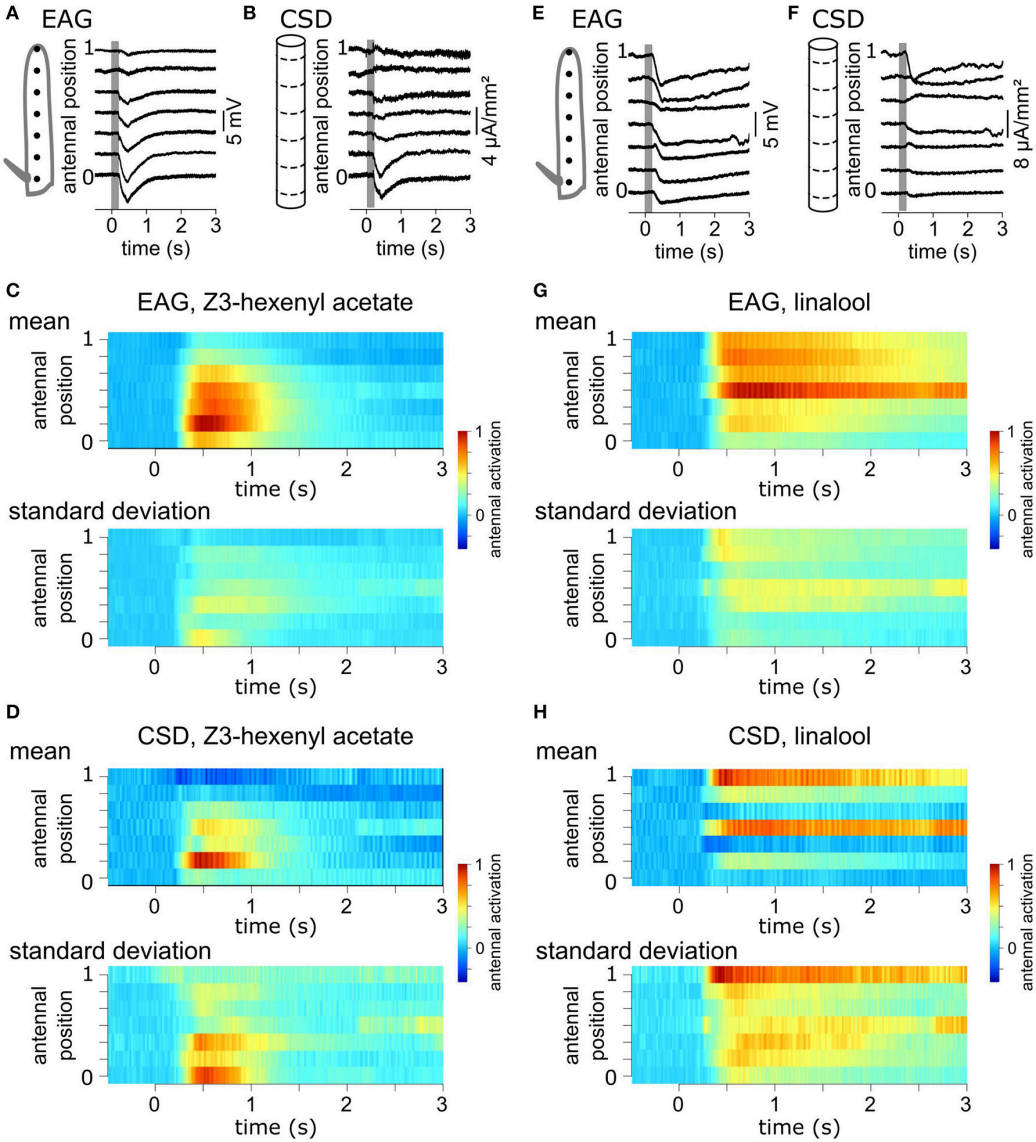
Antennal response maps estimated with the CSD analysis. **(A)** EAGs were recorded in response to puffs of Z3-hexenyl acetate and with the electrode located at seven positions on the funiculus of a *Z. cucurbitae* female along the proximodistal axis. Gray area indicate the stimulation time. Antennal position varied between 0 (proximal position near the arista) and 1 (distal extremity of the funiculus). The scheme in the left represent the funiculus with the position of the electrodes. **(B)** A model of funiculus with seven compartments (scheme on the left) was used to estimate the CSD (right) from the EAG recording shown in **(A)**. **(C)** Spatiotemporal maps of EAG recordings in response to puffs of Z3-hexenyl acetate. Both mean (top) and standard deviation (bottom) are plotted on the same color scale, normalized by the maximum EAG value (*n* = 7 *Z. cucurbitae* females). **(D)** Spatiotemporal maps of CSD response to puffs of Z3-hexenyl acetate. Values were normalized by the maximum CSD value. Same convention as in **(C)**. **(E)** EAG responses of a female *Z. cucurbitae* to a puff of linalool. Same convention as in **(A)**. **(F)** CSD response to a puff of linalool in the individual from **(D)**. Same convention as in **(B)**. **(G)** Spatiotemporal EAG maps of female *Z. cucurbitae* in response to puffs of linalool (*n* = 7). Same convention as in **(C)**. **(H)** Spatiotemporal CSD maps of female *Z. cucurbitae* in response to puffs of linalool (*n* = 7). Same convention as in **(D)**. Data in this figure were included in Jacob et al. (2017b).

A volatile compound activates a subset of the antennal ORNs that are not homogeneously distributed across the surface of the funiculus of the flies. Repeated stimulations of the antenna of *Z. cucurbitae* females with the same compound were applied while the EAG was recorded with an electrode located at different antennal positions. Figure 2A shows EAG responses recorded at seven positions on the antenna to puffs of Z3-hexenyl acetate. The positions were regularly interleaved and ranged along the entire proximo-distal axis of the funiculus surface. The response to Z3-hexenyl acetate was strong in the proximal region of the antenna and decreased gradually down to a small response distally. Then, a CSD model based on the geometry of the funiculus of *Z. cucurbitae* was built. It was used to estimate the spatial distribution of the CSD across the antenna using the EAG data. The antennal response consisted in current sinks (negative peaks of CSD) observed in the proximal third but not in the distal part of the funiculus (Figure 2B). This suggests that the small EAG response recorded with a distal electrode results from the activity of the distant ORNs localized proximally. In the literature, the CSD responses are frequently displayed as a space-time color map. Figures 2C,D shows the mean and standard deviation of response maps to a puff of Z3-hexenyl acetate from a population of seven individuals. The spatial extent of the CSD response is tighter than the spatial extent of the EAG response. Actually, the CSD analysis allows to delineate a sub-region of the funiculus where the activated ORNs lye. Note that the standard deviation is proportionally higher for the CSD map than for the EAG map. Figures 2E–H shows the same protocol performed with puffs of linalool. The EAG response to linalool stimulation was larger distally than proximally, and therefore had a different spatial distribution from the response to Z3-hexenyl acetate stimulation. The CSD analysis revealed two distinct current sinks induced by linalool, one at the distal end and one in the middle region of the funiculus. This suggests that two populations of ORNs are activated by linalool in this species. SSRs would however be necessary to confirm it.

### CSD maps are correlated with functional maps estimated from cellular responses in *Drosophila melanogaster*

**Figure 3:**
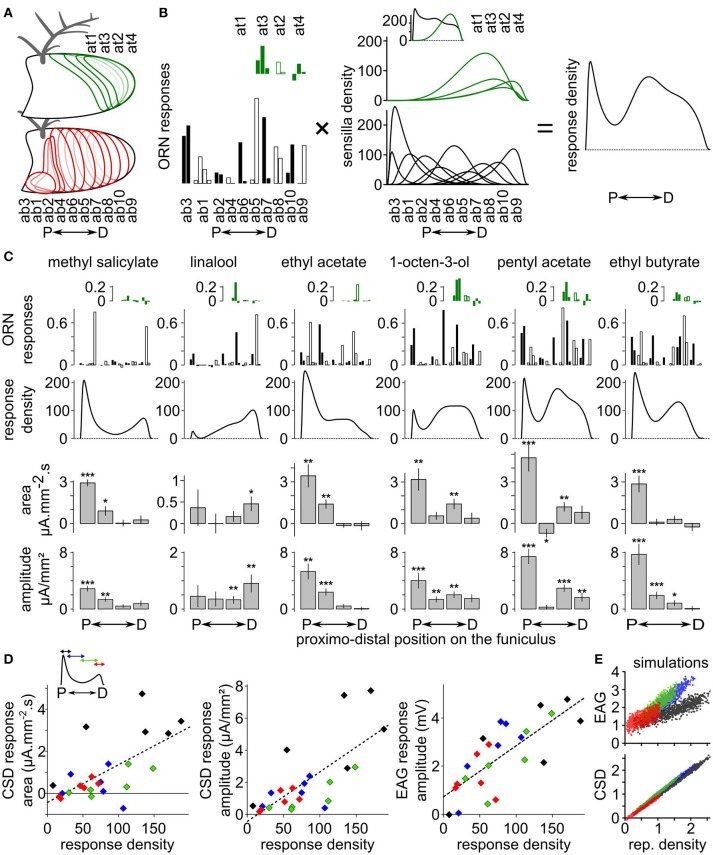
Comparison between CSD maps and cellular maps in *Drosophila melanogaster*. **(A)** Drawings of a funiculus of *D. melanogaster* including the approximative distribution of each type of basiconic (red contours) and trichoid (green contour) sensilla. The name of the sensilla types are ordered from the most proximal to the most distal one (de Bruyne et al., 2001; Lin and Potter, 2015; Grabe et al., 2016). P, proximal; D, distal. **(B)** Method for estimating the response density across the antenna. The response density across the antenna (right) is defined as the sum of the response level of each ORN types (left) multiplied by the spatial density of the corresponding sensilla type (middle). Left, the response levels to a given stimulation are listed in a distal to proximal order. The response levels in consecutive sensilla types are alternatively indicated with filled and empty bars, and each sensilla type include two to four ORNs. Responses of trichoid sensilla (green) are indicated above responses to basiconic sensilla (black). Middle, the sensilla density for each sensilla type was approximated with a logit normal distribution weighted by the number of sensilla on the antenna (Grabe et al., 2016). The units are the number of sensilla per unit of antennal length. A uniform distribution with a value of 100 would mean that the antenna contains 100 sensilla. Inset: total density of basiconic and trichoid sensilla. **(C)** Functional maps in response to six chemicals. The ORN responses (up) were extracted from the database DoOR (Münch and Galizia, 2016) and are expressed in consensual scaled unit. The responses are shown in the same sensilla order as in panel **(B)**. The resulting response densities along the proximo-distal axis are shown below. Down: the bar graphs indicates the area and the amplitude of the CSD response (means + SE, ^***^p < 0.001, ^**^p < 0.01, ^*^p < 0.05, Wilcoxon's signed rank test, *n* = 10). **(D)** Linear correlation between CSD maps and cellular maps. Inset: for each chemical, the curve of response density was averaged over four proximo-distal windows corresponding to the four compartments of the CSD model. The identity of the compartment/window is color coded (from proximal to distal: black, blue, green, and red). The scatterplots show the mean areas and amplitudes of the CSD responses in each antennal compartment and the mean amplitude of the EAG responses in the co-localized electrode in function of the mean response density along the same compartment. Dashed lines: linear regression curves. **(E)** EAG and CSD in function of the response density from simulated datasets. One dot of each color corresponds to one simulated response density built with allocating a random activation level to each basiconic sensilla. Units are arbitrary.

The CSD response maps were compared with functional maps obtained at the cellular level in *D. melanogaster*. EAGs in response to methyl salicylate, linalool, ethyl acetate, 1-octen-3-ol, ethyl butyrate and pentyl acetate were recorded at four antennal positions of 10 *D. melanogaster* females and the CSD responses were calculated. Independently, functional maps in response to the same compounds were estimated using information available on the spatial distribution of each olfactory sensilla types on the antenna (de Bruyne et al., 2001; Lin and Potter, 2015; Grabe et al., 2016), on the number of each olfactory sensilla types (Grabe et al., 2016), and on the response levels of most classes of antennal ORNs extracted from the database DoOR (Münch and Galizia, 2016; Figures 3A,B). Responses of coeloconic sensilla and of at1 sensilla were not on the database, but would likely have a minor impact on the functional maps.

Qualitatively, spatial peaks in the CSD maps were located at the same loci than spatial peaks in the functional maps derived from the cellular responses (Figure 3C). Methyl salicylate induces a cellular response in the proximal part of the antenna. Accordingly, CSD analysis revealed a significant response in the two most proximal compartments (Wilcoxon's signed rank test, respectively *p* < 0.001 and *p* < 0.05). Linalool induces a cellular response in the distal part of the antenna, and CSD analysis revealed a significant response in the most distal compartment only (Wilcoxon's signed rank test *p* < 0.05). The cellular response to ethyl acetate peaks proximally, and significant CSD responses were observed in the two proximal compartments only (Wilcoxon's signed rank test, respectively *p* < 0.01 and *p* < 0.01). 1-octen-3-ol and pentyl acetate induces cellular responses at two antennal loci: a proximal peak, and a second medio-distal peak. Accordingly, significant CSD responses were observed both in the most proximal compartment (Wilcoxon's signed rank test, respectively *p* < 0.01 and *p* < 0.001) and in the second-most distal compartment (Wilcoxon's signed rank test, respectively *p* < 0.01 and *p* < 0.01). Finally, ethyl butyrate induced a cellular response proximally and a significant CSD response was found in the most proximal compartment (Wilcoxon's signed rank test, *p* < 0.001). However, secondary cellular responses induced distally by methyl salicylate and medially by ethyl butyrate were not associated with significant CSD responses. In addition, a significant negative CSD response was observed in the second proximal compartment in response to pentyl acetate (Wilcoxon's signed rank test, *p* < 0.05), while this was not expected from the cellular responses.

Both the area [*F*_(1, 22)_ = 15.53, *p* < 0.001, *r*^2^ = 0.41] and the amplitude [*F*_(1, 22)_ = 24.92, *p* < 0.0001, *r*^2^ = 0.53] of CSD responses were significantly correlated with the cellular response density averaged within the corresponding antennal compartments (Figure 3D). EAGs recorded with electrodes positioned above each compartment also correlated significantly with the mean cellular response density within the compartment [*F*_(1, 22)_ = 29.25, *p* < 0.0001, *r*^2^ = 0.57]. To highlight the difference between EAG and CSD responses, a set of 1,000 response density curves were simulated with attributing a random response level to each basiconic sensilla. These response densities were considered as current source densities, and a CSD model with 100 compartments was used to calculate the resulting EAGs, then another CSD model with four compartments was used to estimate the CSD from four positions of EAGs. Figure 3E shows the relationship between EAGs, CSDs, and response densities in the simulated dataset. Again, both the EAG [*F*_(1, 3998)_ = 4,371, *p* < 10^−15^, *r*^2^ = 0.52] and the CSD [*F*_(1, 3998)_ = 24,870, *p* < 10^−15^, *r*^2^ = 0.98] were significantly correlated with the response density. By construction, the small jitter in the simulated CSD–response density relationship can only be due to the fact that response densities are defined at a smaller spatial scale than the four compartments of the CSD model. It resulted in two unexpected effects: for a response density of 0, the CSD was slightly negative (*p* < 10^−15^) and there was a significant effect of the position of the compartment [*F*_(3, 3995)_ = 3,051, *p* < 10^−15^]. The simulated EAG-response density relationship also depended significantly on the position of the electrode [*F*_(3, 3995)_ = 1,942, *p* < 10^−15^], and the EAG was significantly positive for a subjacent response density of 0 (*p* < 10^−15^). This effect was expected because the activity of distant ORNs contributes additively to the EAG signal.

Similar features were observed in the dataset of CSD responses to the six tested chemicals. Even if not significant, the intersection of the linear regression with a response density of 0 was slightly negative for the area (−0.41 mA·mm^−2^·s, *p* = 0.345) and the amplitude (−0.43 μA·mm^−2^, *p* = 0.462) of CSD responses, and was positive for the EAG responses (0.73 mV, *p* = 0.058; Figure 3D). The effect of the antennal compartment was significant for the area [*F*_(3, 19)_ = 9.0623, *p* < 0.001] and amplitude [*F*_(3, 19)_ = 6.0324, *p* < 0.01] of the CSD responses, but not for the amplitude of the EAG [*F*_(3, 19)_ = 0.7189, *p* = 0.55]. More specifically, CSD responses in the most proximal compartment tended to be larger than in the third compartment for the same level of cellular responses. This effect was directly linked with the nature of the sensilla involved in the cellular response: the most proximal compartment elicited larger amplitudes of CSD responses to 1-octen-3-ol, pentyl acetate and ethyl butyrate than expected from the cellular response, but not for methyl salicylate, linalool and ethyl acetate. The first three compounds activate ab3 sensilla, but not the last three.

As a sum up, the CSD maps correlated with the functional maps derived from the activation of the ORNs. Still, some mismatches arose that might be due to inaccuracy of the electrode or sensilla positioning or inhomogeneous density of olfactory sensilla within the compartments of the CSD model.

### An insufficient sampling of eags biases the CSD estimates

**Figure 4:**
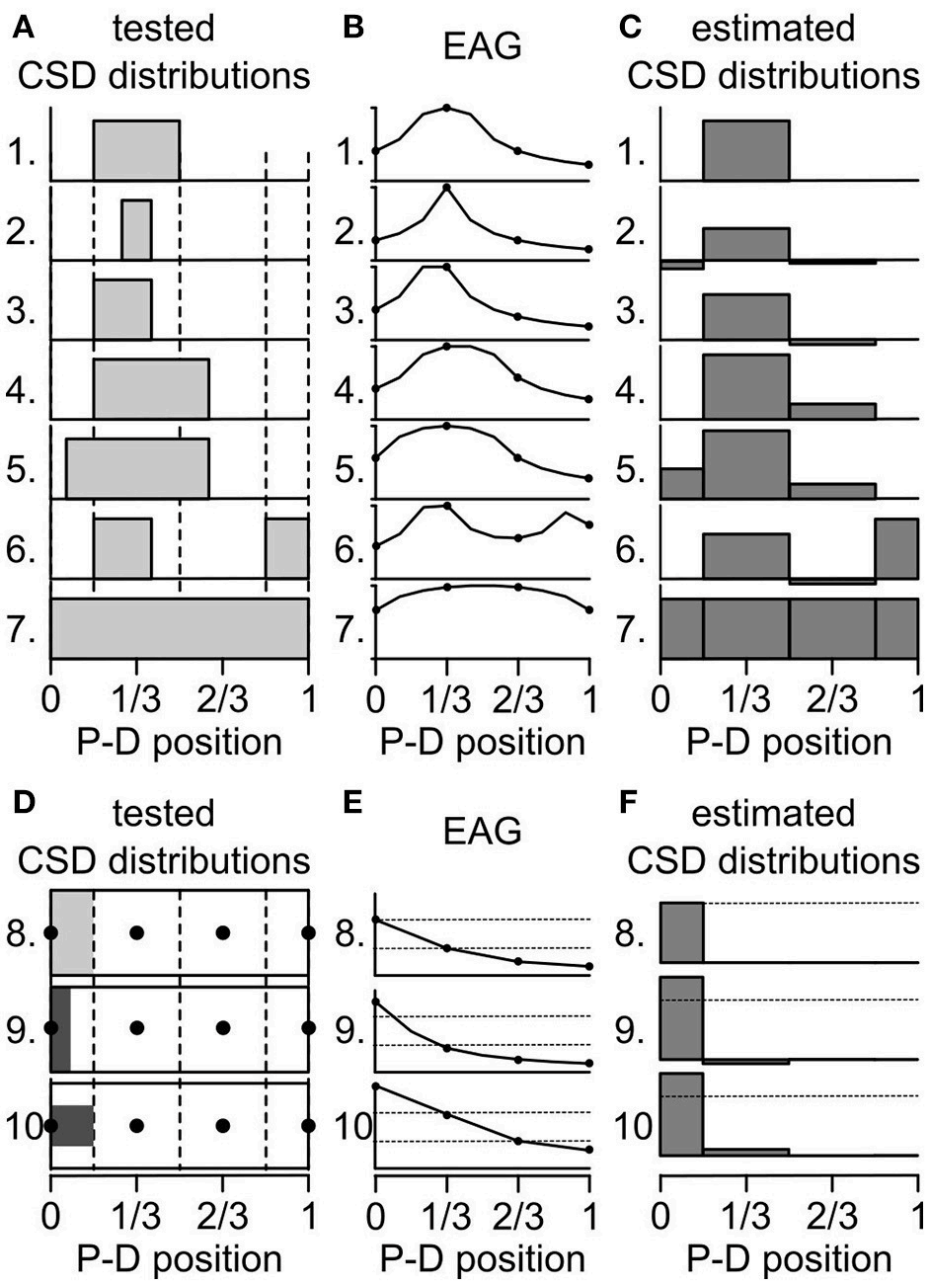
Biased induced by the compartmentalization of the antenna. **(A)** Seven artificial CSD distributions were tested for qualitative biases. At each antennal position along the proximo-distal axis, the CSD was either set to 0 or to 1 (gray bars). The dashed lines delineate the four compartments of an antenna model. P-D, proximodistal. **(B)** The graphs show the EAG distributions that were predicted from the test CSD distributions from **(A)** labeled with the same numbers. The prediction was calculated with a model of antenna with 10 compartments. The amplitude of the EAG was normalized. Only four positions (dots) were used to calculate the CSD. **(C)** The graphs show the CSD distribution that were estimated with a model with four compartments applied on the EAG distributions from panel **(B)**. **(D)** Three additional CSD distributions were tested for quantitative biases. The CSD distributions are shown as unfolded cylinders, the four compartments of the model are delineated by dotted lines and the position of the electrodes are indicated by black dots. The CSD was set to 1 (light gray) in the proximal compartment (8) or to 2 (dark gray) in one half of the proximal compartment: either the proximal half (9) or the central half (10). **(E)** EAG distributions predicted from the test CSD distributions from **(D)**. The amplitudes of the EAG were not normalized. For comparison, dashed lines indicate the amplitudes predicted for the two most proximal electrodes in the distribution number 8. **(F)** CSD distribution estimated from the EAG in **(E)**. For comparison, dashed lines indicate the amplitude of CSD estimated in the proximal compartment in the distribution number 8.

The activated ORNs might be located in regions smaller than one antennal compartment of the antennal model. How would it affect the estimation of the CSD? That question was addressed with testing artificial data sets. First, a test CSD distribution was generated with subdividing the antenna into 10 compartments and the current source was set at 0 or 1 on each compartment. Secondly, a 10-compartment model was used to directly calculate the spatial distribution of EAG induced by the test CSD distribution. Thirdly, a four-compartment model was fed with four positions of the calculated EAG and used to estimate backward the CSD distribution. Finally, the test and the estimated CSD distributions were compared to infer the biases induced by the inappropriate model compartmentalization. Figures 4A–C shows this approach performed on several test CSD distributions. If the location of the activated ORNs matches a compartment of the model, then the estimate is correct (case 1 and 7 in Figure 4). If the activated ORNs are distributed over an area larger than a compartment of the model (case 4 and 5), then the model detects a current source in each compartment that includes active ORNs. If the activated ORNs are distributed in an area smaller than a compartment of the model (cases 2, 3, and 6), then the model detects a current source in this compartment and mistakenly estimates a current source with a reverse polarity in the neighboring compartments. An inhibition of the neighboring compartments would result in the same distribution of EAGs, and therefore both cases cannot be disambiguated with four recording positions.

Next, a quantitative effect was tested with varying the spatial distribution of the same level of current sources within the most proximal compartment out of four (Figures 4D–F). Either the current sources were uniformly distributed within the first compartment with a value of 1 (case 8 in Figure 4), or the current sources lied in half of the proximal compartment only but had a value of 2. This simulates the same number of active ORNs located closer to the proximal electrode. If the current sources were confined to the proximal half of the compartment (case 9 in Figure 4), the EAG in the proximal electrode was increased by 127% compared with case 8, the EAG in the subsequent electrode was decreased to 89%, and the estimated CSD was increased by 136%. If the current sources were confined to the central half of the compartment (case 10 in Figure 4), which was simulated by dividing by two the diameter of the compartment in the test CSD model, the EAG in the proximal electrode was increased by 149% compared with case 8, the EAG in the subsequent electrode was also increased by 200%, and the estimated CSD in the proximal compartment was increased by 136%. These observations can explain some mismatches observed between CSD maps and maps obtained from cellular recordings in *D. melanogaster*. In particular, ab3 sensilla densely fill a proximal region smaller than the first compartment of the antennal model (de Bruyne et al., 2001) that might be located only on the lateral surface of the funiculus where the electrode was set (Grabe et al., 2016). Their activation was associated with a strong CSD response in the proximal compartment and either a significant negative CSD response or an unexpected lack of activation of the subsequent compartment (Figure 3).

### Mapping the antennal response with different sets of electrodes

**Figure 5:**
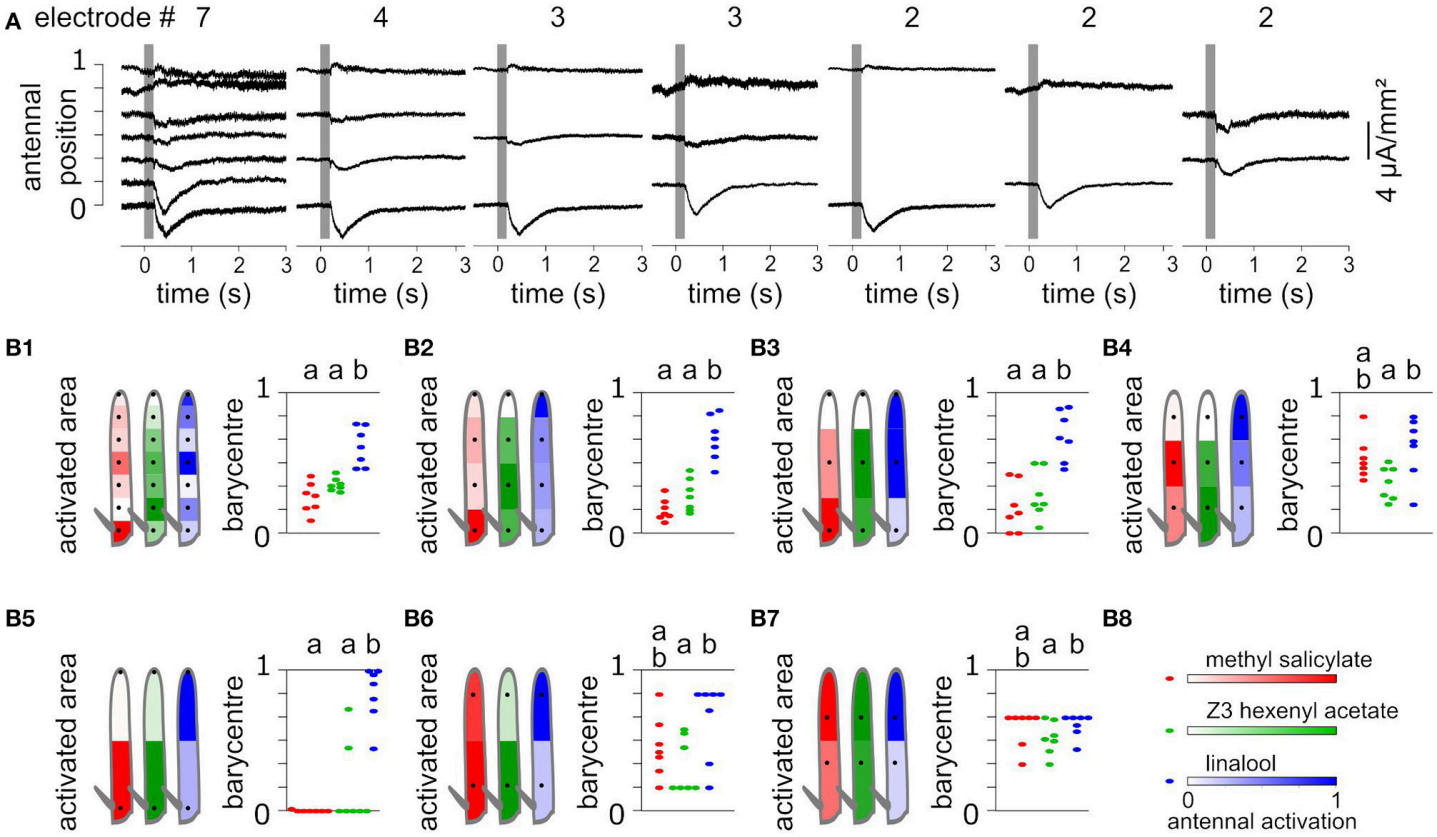
Spatial information collected from different numbers of recordings. **(A)** CSD responses to Z3-hexenyl acetate were estimated from the same individual as in Figures 2A,B. The CSD were estimated from 2, 3, 4, or 7 electrode recording positions. In each case, the CSD responses were horizontally aligned with the considered recording positions. **(B)** Each subpanel **(B1–B7)** shows the CSD responses of *Z. cucurbitae* to puffs of methyl salicylate (red), Z3-hexenyl acetate (green) and linalool (blue) estimated with a different subset of recordings. The subset included seven **(B1)**, four **(B2)**, three **(B3,B4)**, or two **(B5–B7)** recording positions that are indicated with black dots on the drawing of the funiculus (left). The spatial CSD maps were superimposed with the drawing, and the antennal area corresponding to each compartment of the model of the funiculus is color coded with the estimated CSD averaged over seven individuals. Subpanel **(B8)** shows the color scale in normalized CSD units. The scatter plot on the right shows the spatial center of mass of the CSD map calculated for each individual. An artificial jitter was added on the X-axis to distinguish the dots. For each subset of recordings, the center of masses that differ significantly are labeled with a different letter above the plot (paired Wilcoxon's test, *p* < 0.05).

Using a subset of recording positions degrades the localization of evoked CSD responses. To test this effect, EAGs were recorded at seven positions of *Z. cucurbitae* funiculi, and the antenna were stimulated with puffs of methyl salicylate, Z3-hexenyl acetate and linalool. Accordingly, the CSD was estimated along a model of antenna with seven compartments. Methyl salicylate activated the proximal compartment, Z3-hexenyl acetate activated the proximal half of the funiculus with a maximum in the second compartment, and linalool activated mostly the medial and the distal compartments (Figure 5). For each individual, the spatial center of mass of the antennal activation was calculated to approximate the central location of the responding ORNs. It differed significantly between linalool and the other two compounds (paired Wilcoxon test, *p* < 0.05).

Using seven recording positions is barely enough to get an accurate estimate of the position of the activated ORNs in this species, since current sinks were restricted to a single compartment of the model. Nevertheless, lowering the number of recording positions would result in a gain in time and thus increase the number of different stimuli that can be tested within a single individual. To test what spatial information can be inferred from a low number of recordings, the CSDs were estimated again from subsamples of the data and the resulting spatial information was compared to the complete data set. The subsamples included two, three or four positions and were chosen so that the positions were regularly interleaved. In each case the number of compartments of the CSD model matched the number of recording positions, and the length of the modeled antenna was kept identical by extending the first and the last compartment when required. Figure 5 shows the CSD maps estimated in response to puffs of methyl salicylate, Z3-hexenyl acetate and linalool. With four recording positions, most of the spatial features of the responses were still observed, i.e., response to methyl salicylate was maximal in the proximal compartment, response to Z3-hexenyl acetate was maximal in the second compartment, and response to linalool was maximal in the distal compartment. However, the response to linalool was not divided into two distinct current sinks. The response center of masses pointed to the same regions of the funiculus than for the whole data set, and again differed significantly between linalool and the other two odorants (paired Wilcoxon test, *p* < 0.05). For testing three recording positions, the two extremities of the funiculus were either included or not. In both cases, the resulting activation of the antenna by Z3-hexenyl acetate and linalool were observed in the same loci than for the complete data set, and the response center of masses differed significantly between the two stimulations. With methyl salicylate stimulation however, the observed response was located in the same antennal loci than for the complete data set only if the most proximal recording position was included. Finally, while using two recording positions, the centers of mass of the CSD maps were biased toward one of the two positions. Still the spatial structures of the response resembled the one obtained with the complete data set. The response to Z3-hexenyl acetate was localized near the proximal electrode and the response to linalool near the distal electrode and the activation position significantly differed between the two (paired Wilcoxon test on center of masses, *p* < 0.05). The response to methyl salicylate was located proximally only if the most proximal position was considered.

### The modeled geometry of the antenna affects the CSD estimates

**Figure 6:**
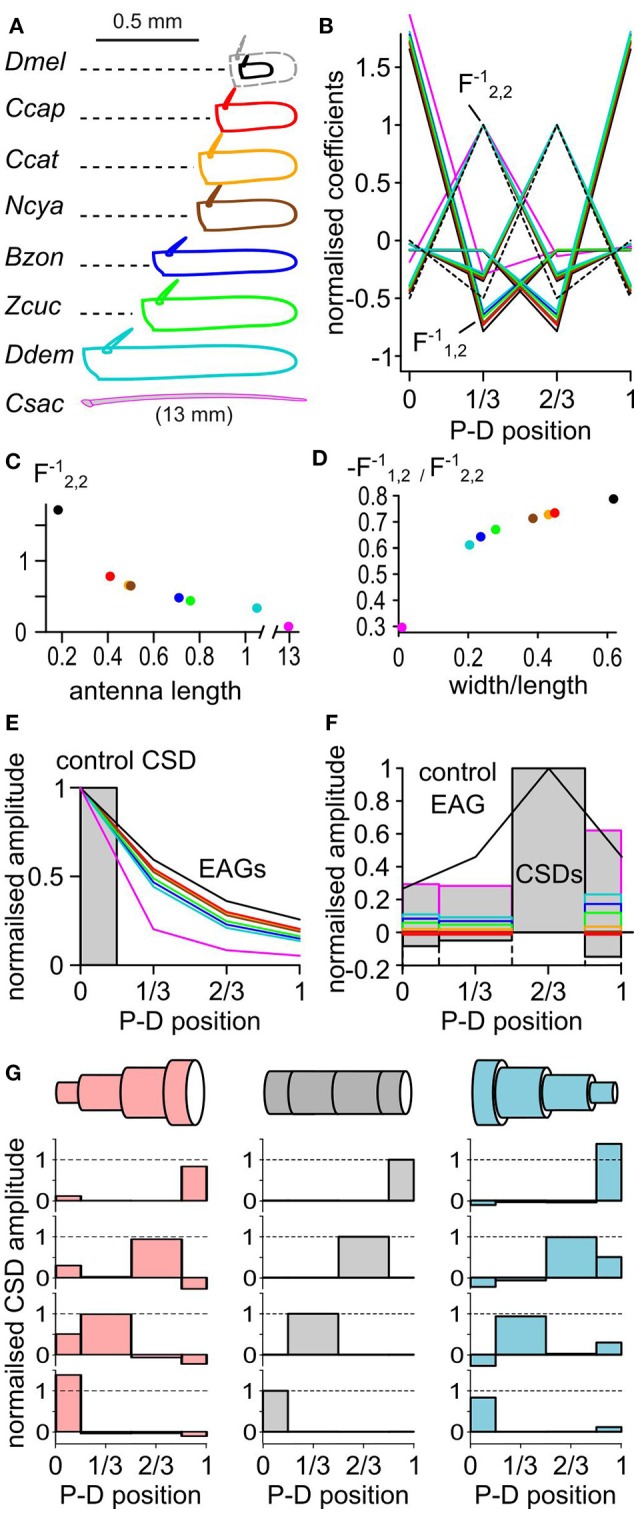
The CSD model depends on the geometry of the antenna. **(A)** The dimensions of the funiculus (length and width) were measured in seven insect species: *D. melanogaster* (*Dmel*), *C. capitata* (*Ccap*), *C. catoirii* (*Ccat*), *N. cyanescens* (*Ncya*), *B. zonata* (*Bzon*), *Z. cucurbitae* (*Zcuc*), and *D. demmerezi* (*Ddem*). The drawing of the funiculus was reshaped to match the average length and width for each species (*n* = 10 per species). Since *D. melanogaster* funiculus is small, it was proportionally enlarged (dotted gray lines) for comparison with the other species. In addition, the length and width of the antenna of a moth *C. sacchariphagus* (*Csac*) was also measured and included. For each panel, colors code for species. P-D, proximodistal. **(B)** Normalized linear coefficients of CSD models with four antennal compartments. Each line links the coefficients for calculating the CSD in one compartment from the four recorded EAGs. For clarity, only half of the coefficients calculated for *C. sacchariphagus* were included. For each line, the maximal coefficient is always for the EAG recorded at the same position as the CSD compartment. The dashed line represents the coefficients used in classical CSD models that had the value of 1, −0.5, or 0 and were calculated only for the compartments 2 and 3. For each CSD model, the coefficient were normalized by $F_{2,2}^{-1}$ that is pinpointed by an arrow. $F_{1,2}^{-1}$ is the coefficient that varies the most between species. **(C)** Scatterplot of the coefficient $F_{2,2}^{-1}$ in function of the antennal length. **(D)** Scatterplot of the ratio between coefficients $F_{1,2}^{-1}$ and $F_{2,2}^{-1}$ in function of the ratio between width and length of the antenna. **(E)** Superimposed EAG distributions (lines) predicted from the same control CSD distribution (boxes) for the different species. **(F)** Superimposed CSD distributions (boxes) estimated from the same EAG distribution (black line) for the different species. **(G)** Three antennal geometries (top) were used to estimate CSDs (bottom) from four different EAG distributions. Each horizontally aligned CSD map is estimated from the same EAG distribution. The antenna is modeled with a cylinder (middle, gray), a cone with a proximo-distal decreasing in diameter (right, light blue), or a cone with a proximo-distal increasing in diameter (left, light red). For each model, the average antennal diameter is the same. In the two conic models, the maximal diameter is three times larger than the minimal diameter.

Classically, the CSD was approximated by the second spatial derivative of the field potential. Hence the CSD could not be estimated at the position of the electrodes in the extremities (Vaknin et al., 1988). With EAG recorded at four positions, the CSD would be estimated only at the second and the third position with the respective linear coefficients (−0.5, 1, −0.5, 0) and (0, −0.5, 1, −0.5). The model used in this study allows the estimation of the CSD at all the electrode positions. The coefficients $F_{\text{ij}}^{-1}$ used to estimate the CSD distribution directly depend on the geometry of the antenna. Thus, the average length and width of the antenna shall be measured and used for calculating the CSD (Figure 1D). The size of the antenna varies between species, and its shape can be elongated or globular. To test the impact of the antennal geometry on the model, the length, width and thickness of the funiculus were measured for 10 individuals in seven fly species, namely *Drosophila melanogaster, Ceratitis capitata, Ceratitis catoirii, Neoceratitis cyanescens, Bactrocera zonata, Zeugodacus cucurbitae*, and *Dacus demmerezi*. The average values were used to model the antenna for each species. In these species, the length of the funiculus varied between 150 and 1,100 μm, and the ratio of width to length varied between 0.2 and 0.6 (Figure 6A). The largest antennae also tended to be the more elongated, with the exception of *Z. cucurbitae* vs. *B. zonata*. An additional antennal model was built from the measurement of the width (125 μm) and the length (13 mm) of the antenna of a female *C. sacchariphagus*.

The coefficients for the different models are shown in Figures 6B–D. The coefficients were larger in the species with the smallest antennae, and the smallest species tend to have higher amplitudes of current source. However, the relative amplitudes of the current sources in the different compartments are not affected by the size of the antenna. On the contrary, the ratio of the width and the length of the funiculus affected the ratios between the different coefficients and therefore impacted the spatial distribution of the estimated CSD. The model calculated for the different species were used to calculate the EAG resulting from the same test distribution of current sources (Figure 6E). Intuitively, the ORNs located proximally should be less likely to polarize the distal end of an elongated funiculus than of a globular one, and this was indeed predicted by the models. Inversely, the models were used to estimate the CSD from a given distribution of EAG signals (Figure 6F). The position of the peak CSD response was always located in the same compartment. The relative amplitude of the CSD in the neighboring compartment varied between −18 and 20% of the peak CSD response.

Even if cylindric antennal model seems reasonable for fruit flies, insect antennae are frequently tapered. A better approximation of the antennal geometry should improve the accuracy of the CSD estimation. CSD distributions were estimated again with three virtual geometries: a cylindrical antenna based on *Z. cucurbitae* dimensions, a conical antenna with a proximo-distal decrease in diameter, and a conical antenna with a proximo-distal increase in diameter (Figure 6G). The average antennal diameter was the same in the three cases, and for the two conical models the largest diameter was three times larger than the smallest one. In each case the peak of the CSD response was located in the same antennal compartment, but its amplitude differed. In the other antennal compartments the CSD response was either positive or negative depending on the model. Generally, the level of CSD response in a compartment was negatively correlated with the surface of the compartment in the model. Thus, a bias due to an approximated geometry of the funiculus would affect marginally the level of CSD response, but should not affect the localization of the CSD peak. For example the CSD maps estimated for *D. melanogaster* had larger responses in the proximal compartment than in the distal ones (Figure 3). This observation might be due to the fact that the basiconic sensilla are sparser distally, but this feature would be less pronounced if a tapered antennal geometry was used.

### Examples of ambiguous CSD estimates

**Figure 7:**
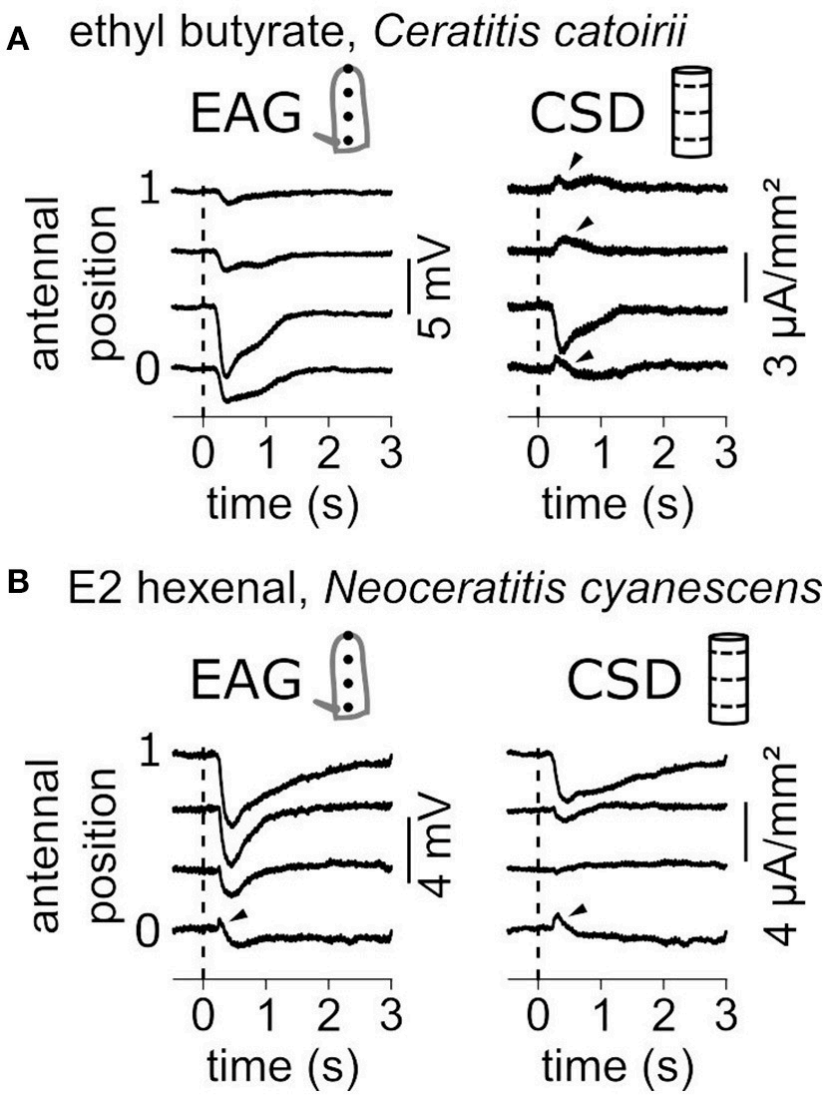
Current sources are occasionally observed. **(A)** Antennal response of a *C. catoirii* female to puffs of ethyl butyrate. Left: The EAG responses were recorded at four antennal positions. The dash line indicated the onset of stimulation. Right: The CSD responses were estimated from the EAG recordings. Apparent current sources are pinpointed with arrowheads. **(B)** Antennal response of a *N. cyanescens* female to puffs of E2 hexenal. Same conventions as in **(A)**. A transient positive response in the EAG signal is also pinpointed.

EAG signals are mostly negative polarizations of the antenna and correspond to current sinks. Figure 7 shows two examples where current sources, i.e., with positive values, were observed. In response to a puff of ethyl butyrate, the funiculus of *C. catoirii* responded with a current sink in the second compartment, and small current sources were also observed in the first, the third and the fourth compartments (Figure 7A). In this case, the current sink is unambiguous, but not the current sources which can result from approximations of the antennal model. Either there is an actual current source, or the current sink covers a subregion of the second compartment of the model, or the geometry of the funiculus was improperly estimated. In response to E2-hexenal, the funiculus of *N. cyanescens* had a current sink in the distal compartment and a current source in the proximal compartment (Figure 7B). In this case, the EAG recorded in the first position also showed a transient positive polarization. This observation cannot result from the CSD model, and therefore it results most likely from an actual current source. As a conclusion, the experimenter should conclude about the existence of a secondary current source only if it is associated with a positive polarization of the antenna observed by EAG.

### Gas-chromatography coupled with CSD analysis

**Figure 8:**
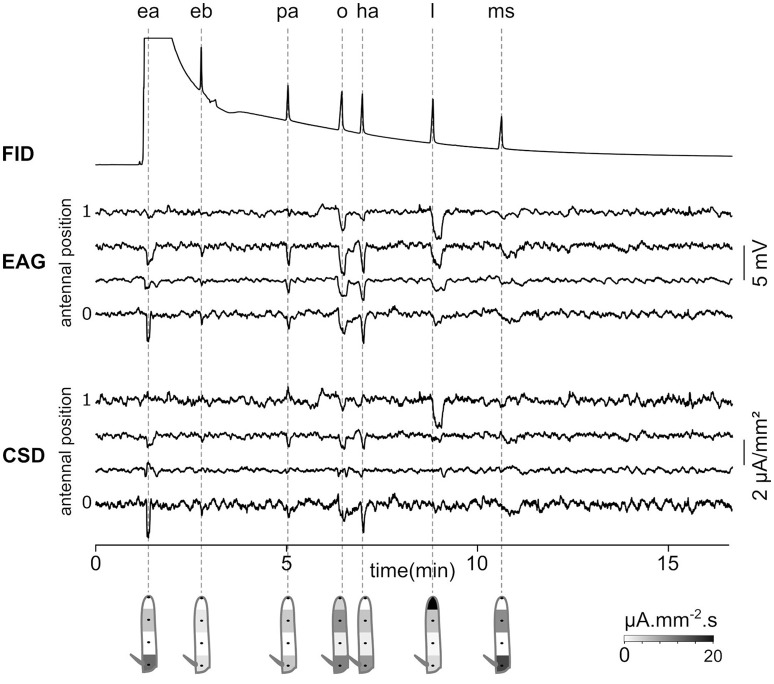
Gas-chromatography coupled with an EAD-CSD. Top: Flame ionization detector (FID) signal recorded at the output of the gas-chromatograph injected with a 10^−3^ dilution of seven compounds in hexane. The compounds are ethyl acetate (ea), ethyl butyrate (eb), pentyl acetate (pa), 1-octen-3-ol (o), Z3-hexenyl acetate (ha), linalool (l), and methyl salicylate (ms). Times of peaks are indicated with dashed lines. Ethyl acetate was ejected at the same time as hexane, and therefore the peak is not visible in the FID signal. Middle: EAG was recorded at four positions of the funiculus of a *Z. cucurbitae* female. Bottom: the CSD response was estimated from EAG recordings. Under each response, the spatial maps were drawn from a temporal integration of CSD responses.

As a proof of concept that CSD analysis of EAG recorded at multiple positions can be used in the research in chemical ecology, EAG recordings were performed in a *Z. cucurbitae* individual while the odorants were provided through a GC. Figure 8 shows the EAG and CSD analysis in response to a mixture of seven synthetic components. Each component was isolated by GC and reached the antenna at a different time. The induced CSD maps revealed an excitation of the proximal compartment by ethyl acetate, Z3 hexenyl acetate and methyl salicylate, an excitation of the third compartment by pentyl acetate and 1-octen-3-ol, and an excitation of the distal compartment by linalool. This result is similar to the results obtained with direct stimulations of the antenna by these compounds (Jacob et al., 2017b). With this experimental setup, the CSD analysis can be used to analyze the insect sensitivity to complex odor mixtures, like the volatile compounds emitted by host plants or by conspecifics.

### CSD mapping of a moth antenna

**Figure 9:**
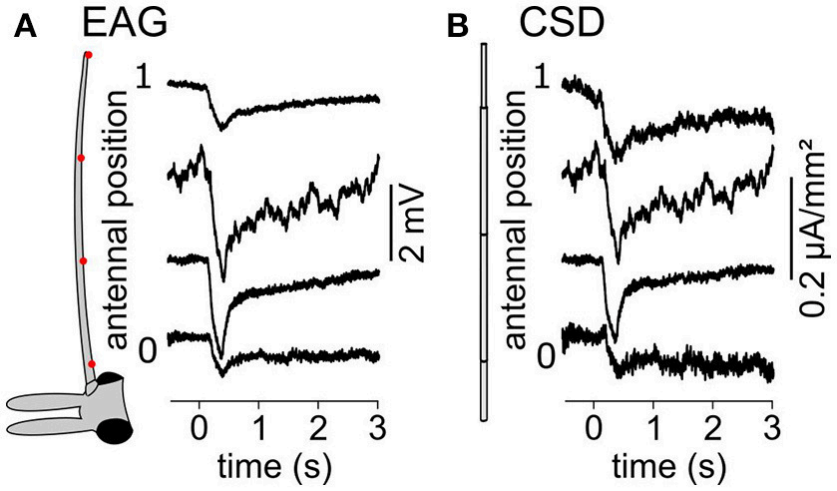
CSD maps of a *C. sacchariphagus* antenna in response to Z3-hexen-1-ol stimulation. **(A)** EAG response was recorded at four antennal positions of the antenna of a female *C. sacchariphagus*. The drawing on the left of the head and one antenna of *C. sacchariphagus* indicates the positions of recording (red dots). **(B)** A four compartments antennal model (left) was used to estimate the CSD response at the four positions (right).

To check if CSD analysis can be used in other insect orders, the EAG was recorded at four positions of the antenna of a female moth *C. sacchariphagus*, the cane sugar stem borer, stimulated with the green leaf volatile Z3-hexen-1-ol. The antenna was modeled with four antennal compartments and the distal one had a smaller diameter to fit the antennal dimension. The resulting CSD map was estimated (Figure 9). CSD responses were observed in each of the four antennal compartments, which amounted, respectively to 0.34, 0.43, 0.55, and 0.50 μA·mm^−2^·s from the proximal to the distal segments. Further repetition of this protocol would be required to confirm that ORNs sensitive to Z3-hexen-1-ol are located all along the antenna of *C. sacchariphagus*, but this observation is in accordance with the current understanding of moth olfactory system which hypothesize that each antennal annulus is a repetition of the same ORNs distribution. In any case, this recording confirms that the CSD approach can also be used in insects with filamentous antenna.

## Discussion

The CSD analysis has been developed to localize the current sources and sinks that cause the field potentials in brain tissues (Mitzdorf, 1985), including the mushroom body in insect brains (Kaulen et al., 1984). This paper shows that the CSD analysis applied to EAG recordings localizes current sinks induced by the olfactory stimulation of the insect antenna. Several indices suggest that the current sinks are co-localized with the activated ORNs. The amplitude of the EAG signal is correlated with the local density of olfactory sensilla at the electrode site (Bigiani et al., 1989; Crnjar et al., 1989). The spatial range of CSD responses to small polar molecules was accordant with the spatial distribution of basiconic sensilla across seven different species (Jacob et al., 2017b). The activation of conserved ORs resulted in conserved positions of current sinks between *D. melanogaster* and tephritids (Jacob et al., 2017b). Finally, this study shows that the maps of CSD responses to six compounds were spatially correlated with functional maps estimated from the number, response level and localization of the different ORNs in *D. melanogaster*.

Recording the EAG at many positions was found necessary to investigate in detail the olfactory response. First, while SSR and single-electrode EAG samples only a fraction of the antennal ORNs (Nagai, 1985; Jacob et al., 2017b), sampling EAG at several positions reveals the activity of most. In a previous paper, the responsiveness of six fruit flies species to seven chemicals broadly distributed in plant odors was tested (Jacob et al., 2017b). With *n* = 10 individuals per species, single EAG revealed a significant response in 67% of the cases while using the four positions of recordings revealed a significant response in 97% of the cases. In addition, the peak amplitude of the CSD response can be used to quantify the olfactory responses as it correlates with the number of responsive ORNs multiplied by their response level. Hence the GC-multiple EAD approach combined with CSD analysis will be useful for chemical ecologists wishing to determine the olfactory sensitivity of an insect to the compounds of a natural blend. Secondly, the CSD analysis complements alternative methods (Hull and Cribb, 2001) suggesting that two chemicals from a blend activate different ORNs, and therefore are not conveying redundant sensory information. Finally the CSD analysis can be used by electrophysiologists to guide further SSR investigation into particular regions of the antenna for species whose olfactory system is not known. In *Drosophila*, functional maps can be explored by combining the exhaustive investigation of all the ORN classes with SSR performed in many insects (Crowley-Gall et al., 2016; Münch and Galizia, 2016) with the estimation of the number of ORNs in a class (Dekker et al., 2006; Grabe et al., 2016), an important property that might be developmentally regulated (Song et al., 2012). Combining SSR with CSD responses might be an interesting alternative for comparing different strains or species in this genus. In *D. melanogaster*, the more distal classes of basiconic sensilla have a sparser spatial distribution (Grabe et al., 2016). Accordingly, higher levels of CSD response were found for proximal ORNs than for distal ORNs with similar activation levels (Figure 3).

### Methodological recommendations for applying CSD analysis to EAG recordings

A couple of potential experimental biases should be considered for the CSD analysis. (1) Each repetition of the same stimulation does not necessarily induce the same neuronal activation. In particular in *ex vivo* preparations the EAG response decreases with time due to a degradation of the biological tissues. Recording simultaneously at multiple antennal positions would improve the CSD estimation, and furthermore result in a gain in time allowing more complex protocols. (2) The recording electrode must be thin enough for having a good spatial resolution. The glass micropipettes I used were 1–2 μm tip diameter. I would not recommend using large electrode contacts with the antenna through a drop of electrolytic gel of liquid for CSD analysis. In addition specific classes of olfactory sensilla might be covered by the electrode in such preparations and not be stimulated. (3) The reference electrode should not be in the vicinity of any olfactory sensilla, otherwise the EAG signal would result from a combination of the evoked potentials recorded by each electrode. Thus, the reference electrode should not be positioned at the base of the antenna but rather in the insect eye or body. (4) The experimenter should be careful about the quality of the electrode contacts and the precision of electrode positioning that might affect the CSD. (5) The model used in this paper hypothesize that the different ORN classes are organized in a 1-dimensionnal grid along the proximo-distal axis. However, some ORNs classes might lay mostly in the borders or the opposite surface of the funiculus. In tephritids, seven or more recording positions are required to estimate specific spatial features of the olfactory response of the antenna. Still, the rough analysis of the CSD resulting from three or four recording positions should be sufficient to localize the antennal activation. In any cases, using a proximal electrode near the arista might be of critical importance since the large basiconic or the clavate sensilla are specifically grouped in this locus both in *Drosophila* (Shanbhag et al., 1999) and in tephritides (Jacob et al., 2017b), while the other sensilla types are more broadly distributed. In this study a correct estimation and localization of *Z. cucurbitae* response to methyl salicylate required indeed a proximal recording.

### The antennal neural tissues are ideal generators of field potentials

Despite of differences due to the propagation of the LFP signal through the brain tissue and of the EAG signal through the insect cuticle, the main principles are similar. A pharmacological disruption of spikes had no effect on the EAG signal (Nagai, 1985; Lucas and Renou, 1992; Nagel and Wilson, 2011), suggesting that the EAG essentially depends on the low frequency dendritic receptor currents as does the LFP. The LFP can be simultaneously recorded with action potentials of the cells within very close distances from the electrode (Roux et al., 2007). Similarly, an SSR electrode records simultaneously the action potentials of the ORNs within the sensillum and the sensillum potentials generated by many ORNs in the surrounding sensilla (Nagai, 1983a). In brain tissues, the difference in spatial range between action potential and field potential recordings is thought to be due to the non-homogeneous properties of the extracellular medium that selectively filters out the high frequency events (Bédard and Destexhe, 2012).

**Figure 10:**
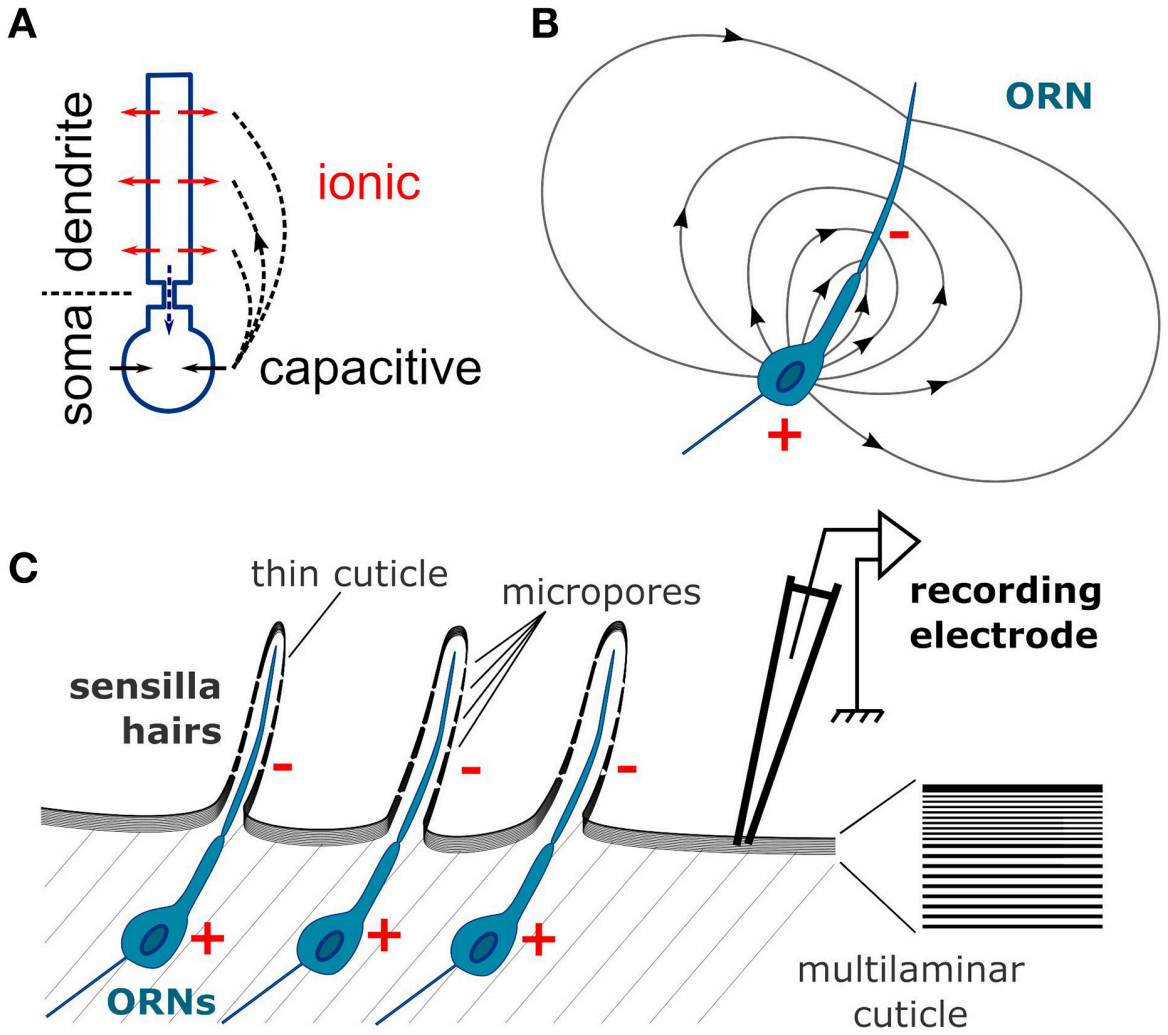
Schematic depiction of the CSD theory applied to the insect antenna. **(A)** Conventional CSD model. Each neuron is depicted as two compartments, dendrite and soma. The neuron is activated via an ionic current (red arrows) in the dendrites that induces a current sink in the extracellular medium. At the same time a capacitive current in the soma (black arrows) induces a current source in the extracellular medium. The current flowing intracellularly between the compartments (dashed blue arrow) is equal and opposite to the current flowing in the extracellular medium (dashed black arrows). **(B)** Electric field generated by an ORN in a homogenously conductive medium. Since the ORN is asymmetric, it results in an electric dipole in the extracellular medium. The single dendrite is the negative pole (−) and the soma is the positive pole (+). **(C)** The organization of the neuronal tissue in the antenna is ideal for generating field potentials. Individual ORNs are perfectly aligned below the cuticle surface. The components of the electric fields perpendicular to the plane of alinement sum up for generating the EAG signal. Right: zoom on the cuticle microstructure. Since the cuticle has a multilaminar organization, it should have an anisotropic conductivity and the electric current should flow in the tangential direction.

The extracellular current sinks and sources are induced by the transmembrane currents. The flow of cations into the dendrites, either due to synaptic activity of central neurons or to receptor activity of the ORN, induces a current sink, while at the same time a reverse capacitive current around the soma induces a current source (Figure 10A; Bazhenov et al., 2011). The neuron can therefore be considered as an electrical dipole (Figure 10B). A direct consequence is that field potentials are negative around the apical dendrites and positive in the vicinity of the soma (Nicholson, 1973). Most olfactory stimulations induce negative deflections of the EAG signal, which were hypothesized to correspond to the dendritic current sinks (Kaissling, 1995). Accordingly, the amplitude of the sensillum potential varies with the position of the electrode and reach a maximal negativity at mid-distance of the apical dendrite (Nagai, 1983a). Positive polarization of the antenna should result from the dendritic current sources that are expected from the odor-evoked inhibition of the constitutive activity of the receptors that triggers orientation behaviors (Cao et al., 2017). However, this study showed that CSD analysis can be misled in reporting inexistent current sources due to an insufficient spatial sampling or to an approximation of the funiculus geometry.

The anatomical characteristics of a neuronal tissue have an impact on the quality of the LFP signal (Mitzdorf, 1985; Buzsáki et al., 2012). The neurons with more asymmetric somato-dendritic architectures generate stronger electrical dipoles upon activation. In addition, if all the neurons are aligned on a plane with the same perpendicular orientation of their dendrites, the contributions of individual neurons to the field potential sum up; if not they cancel each other. Both conditions are optimal in insect antennae explaining the large amplitude of the EAG signals, in particular in *D. melanogaster*. The architecture of an ORN is strictly asymmetric: the single apical dendrite bathes in the sensillum lymph, while the cell body lies below the cuticle. All ORNs are perfectly aligned along the cuticular wall (Figure 10C). Kaissling hypothesized that the EAG signal results from the tangential orientation of the ORN dipoles with negative poles pointing to the distal part of the antenna and positive poles pointing proximally (Kaissling, 1995). However, each distal negative pole cancels out a proximal positive pole of a neighboring ORNs and the resulting signal should be almost zero. Alternatively, only the component of each dipole that is perpendicular to the cuticle surface should contribute to the EAG signal, as was theorized for brain tissues (Mitzdorf, 1985). Nonetheless the insect antenna is an ideal system for the CSD analysis, and has the additional advantage for modeling that the neuronal activity is spatially confined to the geometry of the funiculus surface that is easier to delineate than the geometry of brain tissues.

### How the antennal tissue differs from brain tissues

The classical models of CSD analysis required a uniform resistivity of the extracellular medium in every direction. This is obviously not the case in the external layers of the insect antennae, and the CSD model built in this paper hypothesized instead that the electric current flows in two dimensions along the antennal cuticle and does not penetrate the antenna. This conclusion was brought from two considerations. First, Nagaï bypassed the hemolymph by inserting a tungsten wire all along the antenna of the European corn borer, and this very invasive manipulation did not affect much the EAG properties (Nagai, 1985). Second, in flies the amplitude of the EAG signal drops down as soon as the electrode pierce through the cuticle, despite a decrease of resistance between the recording and reference electrodes due to the contact with the hemolymph. This could be explained if the locations of the current sinks, i.e., the dendrites of the ORNs, are insulated with a high resistivity barrier from the hemolymph but not from the electrode. The cuticle of insects has highly anisotropic mechanical properties (Klocke and Schmitz, 2011; Clark and Triblehorn, 2014), and has hypothetically an anisotropic conductivity as well. Such anisotropy emerges from the laminar microstructure of the insect cuticle (Figure 10C). The multiple layers of chitin and proteins (Vincent and Wegst, 2004; Andrew Jansen et al., 2016), parallel to the cuticular surface, could act as electric insulators that would preclude a perpendicular propagation of current, and would further improve a tangential propagation of current along the inter-laminar medium, a bit like insulation with myelin improves the current propagation along the axon of vertebrate neurons. In addition, the dendritic electric field could be conducted to the outside layers of the cuticle since it is thin on the sensilla and punctured by micropores (Mayo et al., 1987; Stocker, 1994; Shanbhag et al., 1999). Such assumption remains speculative, and I also analyzed the CSD with a 3-dimensional model of electric field which resulted in qualitatively similar results. Another hypothesis is that the resistance is uniform along the entire antennal surface. This might not be the case since the cuticle can be thinner in the distal region of the antenna (Nagai, 1985), so an estimation of the spatial distribution of cuticle conductivity might further improve the accuracy of the CSD model.

### On the use of CSD across the insect orders

In this paper the CSD analysis was used to explore the response properties of species with bulbous antennae from the Diptera order. The CSD analysis revealed a map of olfactory selectivity and sensitivity. The CSD analysis is particularly useful in these species because the antenna includes only one compact olfactory segment, the funiculus. Each class of olfactory sensilla is unevenly distributed on the funiculus surface so that the olfactory sensitivity varies spatially. Recording EAG at multiple positions in moths also revealed localized ORN activity (Nagai, 1981, 1983b, 1985) and this study includes an example of CSD analysis performed in a moth species, thus it can be applied in various species of insects or other arthropods (Machon et al., 2016). The geometry of the modeled antenna should be adapted to each species to accurately estimate CSD and the experimenter might model a tapered antenna if necessary. In moth the micro-architecture of the antenna is more complex than in fruit flies (Sanes and Hildebrand, 1976) which might preclude a comparison of the amplitude of the CSD responses between species. In insects which filamenteous antennae like moths (Sanes and Hildebrand, 1976; Shields and Hildebrand, 2001; Ghaninia et al., 2014) or mosquitoes (Ghaninia et al., 2007), each annulus is thought to be a repetition of the same distribution of ORNs, and accordingly Z3-hexen-1-ol induced a CSD response in all the positions of a *C. sacchariphagus* antenna. Olfactory maps were described at the level of single antennal segment of moths (Ghaninia et al., 2014) but CSD analysis is not usable at this scale. In these species, the CSD analysis can still be used to determine the spatiotemporal patterns of activation of the ORNs that would result from the contact of the antenna with a natural odor plume (Celani et al., 2014). This information is relayed to the processing areas of an insect's brain (Nishino et al., 2018) and could be involved in the searching of the odor source (Lockey and Willis, 2015; Jacob et al., 2017a).

## Conclusion

Most laboratories recorded the EAG with an electrode located at the distal end of the funiculus. Hence the activity of the proximal ORNs was frequently neglected, and part of the olfactory experience of the insect was not known. Occasionally, the EAG was recorded with an electrode capping the stump of the arista (Siciliano et al., 2014) that is located proximally. Recording the EAG in this way samples mostly the activity of the proximal ORNs. In chemical ecology studies, this situation resulted in discrepancies between ecological relevance and EAG responses to chemicals. For example, ethyl acetate, a major fruit compound also found in cuticular extracts, attracts *C. capitata* individuals (Jang et al., 1994; Casaña-Giner et al., 1999). However, this compound elicited only a low EAG response when recorded with a distal electrode (Light et al., 1988, 1992; Jang et al., 1989). This paper describes a method of recording and analysis that allows a deeper exploration of the olfactory sensitivity of insects with globular antennae. The CSD analysis has been extensively used in vertebrate brains and was proven useful in mapping the neuronal activity. This paper presented a model for adapting the CSD analysis to the particularities of the insect antenna. This method revealed for example that ethyl acetate activates actually the proximal region of *C. capitata* antennae (Jacob et al., 2017b), reconciling behavioral and physiological studies. It is a promising tool for future research on the chemical ecology of arthropods.

## Author contributions

VJ conceived and performed the experiments, did the modeling and analysis, and wrote the manuscript.

### Conflict of interest statement

The author declares that the research was conducted in the absence of any commercial or financial relationships that could be construed as a potential conflict of interest.

## References

[B1] Andrew Jansen M. Singh S. S. Chawla N. Franz N. M. (2016). A multilayer micromechanical model of the cuticle of *Curculio longinasus* Chittenden, 1927 (Coleoptera: Curculionidae). J. Struct. Biol. 195, 139–158. 10.1016/j.jsb.2016.05.007 27189867

[B2] Bazhenov M. Lonjers P. Skorheim S. Bedard C. Destexhe A. (2011). Non-homogeneous extracellular resistivity affects the current-source density profiles of up-down state oscillations. Philos. Trans. R. Soc. A Math. Phys. Eng. Sci. 369, 3802–3819. 10.1098/rsta.2011.0119 21893529 PMC3263778

[B3] Bédard C. Destexhe A. (2012). Modeling local field potentials and their interaction with the extracellular medium, in Handbook of Neural Activity Measurement, eds. Brette R. Destexhe A. (Cambridge: Cambridge University Press), 136–191.

[B4] Bhandawat V. Olsen S. R. Gouwens N. W. Schlief M. L. Wilson R. I. (2007). Sensory processing in the *Drosophila* antennal lobe increases reliability and separability of ensemble odor representations. Nat. Neurosci. 10, 1474–1482. 10.1038/nn1976 17922008 PMC2838615

[B5] Biasazin T. D. Karlsson M. F. Hillbur Y. Seyoum E. Dekker T. (2014). Identification of host blends that attract the African invasive fruit fly, *Bactrocera invadens*. J. Chem. Ecol. 40, 966–976. 10.1007/s10886-014-0501-6 25236383

[B6] Bigiani A. Scalera G. Crnjar R. Barbarossa I. T. Magherini P. C. Pietra P. (1989). Distribution and function of the antennal olfactory sensilla in *Ceratitis capitata* Wied. (Diptera, Trypetidae). Bol. di Zool. 56, 305–311. 10.1080/11250008909355655

[B7] Buzsáki G. Anastassiou C. A. Koch C. (2012). The origin of extracellular fields and currents-EEG, ECoG, LFP and spikes. Nat. Rev. Neurosci. 13, 407–420. 10.1038/nrn3241 22595786 PMC4907333

[B8] Cao L. H. Yang D. Wu W. Zeng X. Jing B. Y. Li M. T.. (2017). Odor-evoked inhibition of olfactory sensory neurons drives olfactory perception in *Drosophila*. Nat. Commun. 8, 1–13. 10.1038/s41467-017-01185-0 29116083 PMC5676773

[B9] Casaña-Giner V. Gandía-Balaguer A. Primo-Yúfera E. (1999). Field trial of an attractant mixture for dipterous, including the pest *Ceratitis capitata* (Wiedemann) (Dipt., Tephritidae), in Valencia, Spain. J. Appl. Entomol. 123, 47–48. 10.1046/j.1439-0418.1999.00329.x

[B10] Celani A. Villermaux E. Vergassola M. (2014). Odor landscapes in turbulent environments. Phys. Rev. X 4, 041015 10.1103/PhysRevX.4.041015

[B11] Clark A. J. Triblehorn J. D. (2014). Mechanical properties of the cuticles of three cockroach species that differ in their wind-evoked escape behavior. PeerJ 2, e501. 10.7717/peerj.501 25101230 PMC4121590

[B12] Clavijo Mccormick A. C. Gershenzon J. Unsicker S. B. (2014). Little peaks with big effects: establishing the role of minor plant volatiles in plant–insect interactions. Plant Cell Environ. 37, 1836–1844. 10.1111/pce.12357 24749758

[B13] Couto A. Alenius M. Dickson B. J. (2005). Molecular, anatomical, and functional organization of the *Drosophila* olfactory system. Curr. Biol. 15, 1535–1547. 10.1016/j.cub.2005.07.034 16139208

[B14] Crnjar R. Scalera G. Liscia A. Angioy A. M. Bigiani A. Pietra P. (1989). Morphology and EAG mapping of the antennal olfactory receptors in *Dacus oleae*. Entomol. Exp. Appl. 51, 77–85. 10.1111/j.1570-7458.1989.tb01216.x

[B15] Crowley-Gall A. Date P. Han C. Rhodes N. Andolfatto P. Layne J. E.. (2016). Population differences in olfaction accompany host shift in *Drosophila mojavensis*. Proc. R. Soc. London Ser. B-Biological Sci. 283, 20161562. 10.1098/rspb.2016.1562 27581882 PMC5013806

[B16] de Bruyne M. Foster K. Carlson J. R. (2001). Odor coding in the *Drosophila* antenna. Neuron 30, 537–552. 10.1016/S0896-6273(01)00289-6 11395013

[B17] Dekker T. Ibba I. Siju K. P. Stensmyr M. C. Hansson B. S. (2006). Olfactory shifts parallel superspecialism for toxic fruit in *Drosophila melanogaster* sibling, *D. sechellia*. Curr. Biol. 16, 101–109. 10.1016/j.cub.2005.11.075 16401429

[B18] Duyck P. F. Quilici S. (2002). Survival and development of different life stages of three *Ceratitis* spp. (Diptera: Tephritidae) reared at five constant temperatures. Bull. Entomol. Res. 92, 461–469. 10.1079/BER2002188 17598297

[B19] Dweck H. K. Ebrahim S. A. Khallaf M. A. Koenig C. Farhan A. Stieber R.. (2016). Olfactory channels associated with the *Drosophila* maxillary palp mediate short- and long-range attraction. Elife 5, e14925. 10.7554/eLife.14925 27213519 PMC4927298

[B20] Dweck H. K. Ebrahim S. A. Kromann S. Bown D. Hillbur Y. Sachse S.. (2013). Olfactory preference for egg laying on citrus substrates in *Drosophila*. Curr. Biol. 23, 2472–2480. 10.1016/j.cub.2013.10.047 24316206

[B21] Dweck H. K. Ebrahim S. A. M. Farhan A. Hansson B. S. Stensmyr M. C. (2015). Olfactory proxy detection of dietary antioxidants in *Drosophila*. Curr. Biol. 25, 455–466. 10.1016/j.cub.2014.11.062 25619769

[B22] Einzelnen A. N. (1962). Elektrophysiologische untersuchungen an einzelnen geruchsrezeptoren auf den antennen des Totengrabers (Necrophorus, Coleoptera). Z. Vgl. Physiol. 46, 212–248. 10.1007/BF00341551

[B23] Ghaninia M. Ignell R. Hansson B. S. (2007). Functional classification and central nervous projections of olfactory receptor neurons housed in antennal trichoid sensilla of female yellow fever mosquitoes, *Aedes aegypti*. Eur. J. Neurosci. 26, 1611–1623. 10.1111/j.1460-9568.2007.05786.x 17880395 PMC2121139

[B24] Ghaninia M. Olsson S. B. Hansson B. S. (2014). Physiological organization and topographic mapping of the antennal olfactory sensory neurons in female hawkmoths, *Manduca sexta*. Chem. Senses 39, 655–671. 10.1093/chemse/bju037 25092901

[B25] Grabe V. Baschwitz A. Dweck H. K. M. Lavista-Llanos S. Hansson B. S. Sachse S. (2016). Elucidating the neuronal architecture of olfactory glomeruli in the *Drosophila* antennal lobe. Cell Rep. 16, 3401–3413. 10.1016/j.celrep.2016.08.063 27653699

[B26] Hull C. D. Cribb B. W. (2001). Olfaction in the queensland fruit fly, *Bactrocera tryoni*. I: Identification of olfactory receptor neuron types responding to environmental odors. J. Chem. Ecol. 27, 871–887. 10.1023/A:1010374617409 11471941

[B27] Jacob V. Monsempès C. Rospars J. P. Masson J. B. Lucas P. (2017a). Olfactory coding in the turbulent realm. PLoS Comput. Biol. 13:e1005870. 10.1371/journal.pcbi.1005870 29194457 PMC5736211

[B28] Jacob V. Scolari F. Delatte H. Gasperi G. Jacquin-Joly E. Malacrida A. R.. (2017b). Current source density mapping of antennal sensory selectivity reveals conserved olfactory systems between tephritids and *Drosophila*. Sci. Rep. 7, 15304. 10.1038/s41598-017-15431-4 29127313 PMC5681579

[B29] Jang E. B. Light D. M. Binder R. G. Flath R. A. Carvalho L. A. (1994). Attraction of female mediterranean fruit flies to the five major components of male-produced pheromone in a laboratory flight tunnel. J. Chem. Ecol. 20, 9–20. 10.1007/BF02065987 24241695

[B30] Jang E. B. Light D. M. Flath R. A. Nagata J. T. Mon T. R. (1989). Electroantennogram responses of the Mediterranean fruit fly, *Ceratitis capitata* to identified volatile constituents from calling males. Entomol. Exp. Appl. 50, 7–19. 10.1111/j.1570-7458.1989.tb02307.x

[B31] Joseph R. M. Carlson J. R. (2015). *Drosophila* chemoreceptors: a molecular interface between the chemical world and the brain. Trends Genet. 31, 683–695. 10.1016/j.tig.2015.09.005 26477743 PMC4674303

[B32] Kaissling K.-E. (1995). Single unit and electroantennogram recordings in insect olfactory organs, in Experimental Cell Biology of Taste and Olfaction, eds. Spielman A. Brand J. (Boca Raton, FL: CRC Press), 361–377.

[B33] Kaissling K. E. (1986). Chemo-electrical transduction in insect olfactory receptors. Annu. Rev. Neurosci. 9, 121–145. 10.1146/annurev.ne.09.030186.001005 3518584

[B34] Kaulen P. Erber J. Mobbs P. (1984). Current source-density analysis in the mushroom bodies of the honeybee (*Apis mellifera carnica*). J. Comp. Physiol. A 154, 569–582. 10.1007/BF00610170

[B35] Klocke D. Schmitz H. (2011). Water as a major modulator of the mechanical properties of insect cuticle. Acta Biomater. 7, 2935–2942. 10.1016/j.actbio.2011.04.004 21515418

[B36] Light D. M. Jang E. B. Dickens J. C. (1988). Electroantennogram responses of the mediterranean fruit fly, *Ceratitis capitata*, to a spectrum of plant volatiles. J. Chem. Ecol. 14, 159–180. 10.1007/BF01022539 24277002

[B37] Light D. M. Jang E. B. Flath R. A. (1992). Electroantennogram responses of the Mediterranean fruit fly, *Ceratitis capitata*, to the volatile constituents of nectarines. Entomol. Exp. Appl. 63, 13–26. 10.1111/j.1570-7458.1992.tb02415.x

[B38] Lin C. C. Potter C. J. (2015). Re-classification of *Drosophila melanogaster* trichoid and intermediate sensilla using fluorescence-guided single sensillum recording. PLoS ONE 10:e0139675. 10.1371/journal.pone.0139675 26431203 PMC4592000

[B39] Lockey J. K. Willis M. A. (2015). One antenna, two antennae, big antennae, small: total antennae length, not bilateral symmetry, predicts odor-tracking performance in the American cockroach *Periplaneta americana*. J. Exp. Biol. 218, 2156–2165. 10.1242/jeb.117721 25987729

[B40] Lucas P. Renou M. (1992). Electrophysiological study of the effects of deltamethrin, bioresmethrin, and DDT on the activity of pheromone receptor neurones in two moth species. Pestic. Biochem. Physiol. 43, 103–115. 10.1016/0048-3575(92)90024-T

[B41] Machon J. Ravaux J. Zbinden M. Lucas P. (2016). New electroantennography method on a marine shrimp in water. J. Exp. Biol. 219, 3696–3700. 10.1242/jeb.140947 27638619

[B42] Mansourian S. Corcoran J. Enjin A. Löfstedt C. Dacke M. Stensmyr M. C. (2016). Fecal-derived phenol induces egg-laying aversion in *Drosophila*. Curr. Biol. 26, 2762–2769. 10.1016/j.cub.2016.07.065 27641770

[B43] Mayo I. Anderson M. Burguete J. Robles Chillida E. (1987). Structure of superficial chemoreceptive sensilla on the third antennal segment of *Ceratitis capitata* (Wiedemann) (Diptera; Tephritidae). Int. J. Insect Morphol. Embryol. 16, 131–141. 10.1016/0020-7322(87)90013-4

[B44] Mitzdorf U. (1985). Current source-density method and application in cat cerebral cortex: investigation of evoked potentials and EEG phenomena. Physiol. Rev. 65, 37–100. 10.1152/physrev.1985.65.1.37 3880898

[B45] Münch D. Galizia C. G. (2016). DoOR 2. 0–Comprehensive mapping of *Drosophila melanogaster* odorant responses. Sci. Rep. 6, 21841 10.1038/srep21841 26912260 PMC4766438

[B46] Nagai T. (1981). Electroantennogram response gradient on the antenna of the European corn borer, *Ostrinia nubilalis*. J. Insect Physiol. 27, 889–894. 10.1016/0022-1910(81)90090-1

[B47] Nagai T. (1983a). On the relationship between the electroantennogram and simultaneously recorded single sensillum response of the European corn borer, *Ostrinia nubilalis*. Arch. Insect Biochem. Physiol. 1, 85–91. 10.1002/arch.940010109

[B48] Nagai T. (1983b). Spread of local electroantennogram response of the European corn borer, *Ostrinia nubilalis*. Pestic. Biochem. Physiol. 19, 291–298. 10.1016/0048-3575(83)90057-3

[B49] Nagai T. (1985). Summation and gradient characteristics of local electroantennogram response of the European corn borer, *Ostrinia nubilalis*. Pestic. Biochem. Physiol. 24, 32–39. 10.1016/0048-3575(85)90110-5

[B50] Nagel K. I. Wilson R. I. (2011). Biophysical mechanisms underlying olfactory receptor neuron dynamics. Nat. Neurosci. 14, 208–216. 10.1038/nn.2725 21217763 PMC3030680

[B51] Nicholson C. (1973). Theoretical analysis of field potentials in anisotropic ensembles of neuronal elements. IEEE Trans. Biomed. Eng. 20, 278–288. 10.1109/TBME.1973.324192 4708762

[B52] Nishino H. Iwasaki M. Paoli M. Kamimura I. Yoritsune A. Mizunami M. (2018). Spatial receptive fields for odor localization. Curr. Biol. 28, 600–608.e3. 10.1016/j.cub.2017.12.055 29429617

[B53] Nowotny T. de Bruyne M. Berna A. Z. Warr C. G. Trowell S. C. (2014). *Drosophila* olfactory receptors as classifiers for volatiles from disparate real world applications. Bioinspir. Biomim. 9, 046007. 10.1088/1748-3182/9/4/046007 25313522

[B54] Olsson S. B. Hansson B. S. (2013). Electroantennogram and single sensillum recording in insect antennae, in Methods in Molecular Biology, vol. 1068, ed. Touhara K. (Berlin: Springer Science; Business Media, LLC), 157–177. 10.1007/978-1-62703-619-1_11 24014360

[B55] Olsson S. B. Linn C. E. Roelofs W. L. (2006). The chemosensory basis for behavioral divergence involved in sympatric host shifts. II: olfactory receptor neuron sensitivity and temporal firing pattern to individual key host volatiles. J. Comp. Physiol. A 192, 289–300. 10.1007/s00359-005-0066-5 16315070

[B56] Pettersen K. H. Devor A. Ulbert I. Dale A. M. Einevoll G. T. (2006). Current-source density estimation based on inversion of electrostatic forward solution: effects of finite extent of neuronal activity and conductivity discontinuities. J. Neurosci. Methods 154, 116–133. 10.1016/j.jneumeth.2005.12.005 16436298

[B57] Pitts W. (1952). Investigations on synaptic transmission, in Cybernetics, Trans. 9th Conf. Josiah Macy Foundation, ed. von Foerster H. (New York, NY: Diaphanes), 159–166.

[B58] Potworowski J. Jakuczun W. Łeski S. Wójcik D. K. (2012). Kernel current source density method. Neural Comput. 24, 541–575. 10.1162/NECO_a_00236 22091662

[B59] Ramanujan S. (1914). Modular equations and approximations to pi. Q. J. Pure Appl. Math. 45, 350–372.

[B60] Ronderos D. S. Lin C. C. Potter C. J. Smith D. P. (2014). Farnesol-detecting olfactory neurons in *Drosophila*. J. Neurosci. 34, 3959–3968. 10.1523/JNEUROSCI.4582-13.2014 24623773 PMC3951695

[B61] Rospars J. P. Grémiaux A. Jarriault D. Chaffiol A. Monsempes C. Deisig N.. (2014). Heterogeneity and convergence of olfactory first-order neurons account for the high speed and sensitivity of second-order neurons. PLoS Comput. Biol. 10:e1003975. 10.1371/journal.pcbi.1003975 25474026 PMC4256018

[B62] Roux S. G. Cenier T. Garcia S. Litaudon P. Buonviso N. (2007). A wavelet-based method for local phase extraction from a multi-frequency oscillatory signal. J. Neurosci. Methods 160, 135–143. 10.1016/j.jneumeth.2006.09.001 17049617

[B63] Sanes J. R. Hildebrand J. G. (1976). Structure and development of antennae in a moth, *Manduca sexta*. Dev. Biol. 51, 282–299. 10.1016/0012-1606(76)90144-5 955260

[B64] Schneider D. (1957a). Electrophysiological investigation on the antennal receptors of the silk moth during chemical and mechanical stimulation. Experientia 13, 89–91.13414779

[B65] Schneider D. (1957b). Elektrophysiologische untersuchungen von chemo-und mechanorezeptoren der antenne des seidenspinners *Bombyx mori* L. Z. Vgl. Physiol. 40, 8–41.

[B66] Schneider D. Boeckh J. (1962). Rezeptorpotential und nervenimpulse einzelner olfaktorischer sensillen der insektenantenne. Z. Vgl. Physiol. 45, 405–412. 10.1007/BF00340462

[B67] Shanbhag S. Müller B. Steinbrecht R. (1999). Atlas of olfactory organs of Drosophila melanogaster 1. Types, external organization, innervation and distribution of olfactory sensilla. Int. J. Insect Morphol. Embryol. 28, 377–397. 10.1016/S0020-7322(99)00039-2

[B68] Shields V. D. Hildebrand J. G. (2001). Recent advances in insect olfaction, specifically regarding the morphology and sensory physiology of antennal sensilla of the female sphinx moth *Manduca sexta*. Microsc. Res. Tech. 55, 307–329. 10.1002/jemt.1180 11754510 PMC2386875

[B69] Siciliano P. He X. L. Woodcock C. Pickett J. A. Field L. M. Birkett M. A.. (2014). Identification of pheromone components and their binding affinity to the odorant binding protein CcapOBP83a-2 of the Mediterranean fruit fly, *Ceratitis capitata*. Insect Biochem. Mol. Biol. 48, 51–62. 10.1016/j.ibmb.2014.02.005 24607850 PMC4003389

[B70] Song E. de Bivort B. Dan C. Kunes S. (2012). Determinants of the *Drosophila* odorant receptor pattern. Dev. Cell 22, 363–376. 10.1016/j.devcel.2011.12.015 22340498

[B71] Stensmyr M. C. Dweck H. K. Farhan A. Ibba I. Strutz A. Mukunda L.. (2012). A conserved dedicated olfactory circuit for detecting harmful microbes in *Drosophila*. Cell 151, 1345–1357. 10.1016/j.cell.2012.09.046 23217715

[B72] Stocker R. F. (1994). The organization of the chemosensory cystem in *Drosophila melanogaster*: a review. Cell Tissue Res. 275, 3–26. 10.1007/BF00305372 8118845

[B73] Tait C. Batra S. Ramaswamy S. S. Feder J. L. Olsson S. B. (2016). Sensory specificity and speciation: a potential neuronal pathway for host fruitodour discrimination in *Rhagoletis pomonella*. Proc. R. Soc. London Ser. B Biol. Sci. 283, 1–9. 10.1098/rspb.2016.2101 28003447 PMC5204164

[B74] Vaknin G. DiScenna P. G. Teyler T. J. (1988). A method for calculating current source density (CSD) analysis without resorting to recording sites outside the sampling volume. J. Neurosci. Methods 24, 131–135. 10.1016/0165-0270(88)90056-8 3405010

[B75] Vincent J. F. Wegst U. G. (2004). Design and mechanical properties of insect cuticle. Arthropod. Struct. Dev. 33, 187–199. 10.1016/j.asd.2004.05.006 18089034

